# GFAP isoforms control intermediate filament network dynamics, cell morphology, and focal adhesions

**DOI:** 10.1007/s00018-016-2239-5

**Published:** 2016-05-03

**Authors:** Martina Moeton, Oscar M. J. A. Stassen, Jacqueline A. Sluijs, Vincent W. N. van der Meer, Liselot J. Kluivers, Hedde van Hoorn, Thomas Schmidt, Eric A. J. Reits, Miriam E. van Strien, Elly M. Hol

**Affiliations:** 1 Netherlands Institute for Neuroscience, Royal Netherlands Academy of Arts and Sciences, Amsterdam, The Netherlands; 2 Department of Translational Neuroscience, Brain Center Rudolf Magnus, University Medical Center Utrecht, Universiteitsweg 100, 3584 CG Utrecht, The Netherlands; 3 Physics of Life Processes, Leiden Institute of Physics, Leiden, The Netherlands; 4 Cell Biology and Histology, AMC Medical Center, Amsterdam, The Netherlands; 5 Swammerdam Institute for Life Sciences, Center for Neuroscience, University of Amsterdam, Amsterdam, The Netherlands; 6 Soft Tissue Biomechanics & Engineering, Department of biomedical engineering, Eindhoven University of Technology, Eindhoven, The Netherlands

**Keywords:** GFAP, Astrocytoma, FRAP, Intermediate filaments

## Abstract

Glial fibrillary acidic protein (GFAP) is the characteristic intermediate filament (IF) protein in astrocytes. Expression of its main isoforms, GFAPα and GFAPδ, varies in astrocytes and astrocytoma implying a potential regulatory role in astrocyte physiology and pathology. An IF-network is a dynamic structure and has been functionally linked to cell motility, proliferation, and morphology. There is a constant exchange of IF-proteins with the network. To study differences in the dynamic properties of GFAPα and GFAPδ, we performed fluorescence recovery after photobleaching experiments on astrocytoma cells with fluorescently tagged GFAPs. Here, we show for the first time that the exchange of GFP–GFAPδ was significantly slower than the exchange of GFP–GFAPα with the IF-network. Furthermore, a collapsed IF-network, induced by GFAPδ expression, led to a further decrease in fluorescence recovery of both GFP–GFAPα and GFP–GFAPδ. This altered IF-network also changed cell morphology and the focal adhesion size, but did not alter cell migration or proliferation. Our study provides further insight into the modulation of the dynamic properties and functional consequences of the IF-network composition.

**Electronic supplementary material:**

The online version of this article (doi:10.1007/s00018-016-2239-5) contains supplementary material, which is available to authorized users.

## Introduction

Intermediate filaments (IFs) are part of the cytoskeleton. Together with actin filaments and microtubules, they form an integrated system that regulates many cellular processes, such as cell morphology, cell signaling, cell migration, and proliferation [1–4]. The main IF protein expressed in astrocytes is glial fibrillary acidic protein (GFAP). The ten different GFAP isoforms, of which GFAPα is the canonical isoform, are formed by alternative splicing [5, 6]. The function of GFAP and its isoforms is still elusive, but there is emerging evidence that at least one isoform, GFAPδ, alters the properties of the IF network. GFAPδ differs from GFAPα only in its C-terminal tail, and in non-pathological human brains, and is expressed in specific types of astrocytes, including the adult neural stem cells in the human subventricular zone and subpial astrocytes [7–9]. The GFAPδ protein has a unique 41 amino acids long C-terminal tail [6, 7] and is one amino acid shorter than the canonical GFAPα protein [10]. In pathological conditions, GFAPδ is expressed in certain types of reactive gliosis and glial tumors [11–15]. The tail of GFAPδ disables the protein to form homodimers making it impossible to self-assemble [16]. GFAPδ is able to form heterodimers with other type III IF proteins and can, therefore, be integrated in an IF network. Depending on the level of expression and the concentration of other IFs present, GFAPδ is either tolerated in the network or it causes the whole IF network to collapse in the perinuclear region [7, 17]. Assembly experiments in a cell free environment showed that GFAP networks start to collapse when there is more than 10 % of GFAPδ protein present in the network [17].

In the cell, IF proteins are present in a soluble form in the cytoplasm and in filamentous structures that form an important part of the cell’s cytoskeleton [18, 19]. These IF networks are highly motile structures that are constantly rearranged. The proteins within the filaments are also dynamic, since there is an active exchange between the filamentous and non-filamentous pool of IF proteins [20–23]. IF networks that are already formed can be actively disassembled by phosphorylation of IF proteins, whereas the lack of dephosphorylation will hamper new IF network assembly [24, 25]. It has been shown that phosphorylation of GFAP at the N-terminal head domain by kinases, such as Aurora B or CF kinase, is important for proper dissociation from the filaments during cytokinesis [25–28].

Previously, we showed both in vitro and in vivo that physiological levels of GFAPδ are well tolerated in a GFAPα network [7, 8, 15, 17], although it has also been shown in a cell free system and in vitro that a high expression of GFAPδ can lead to an IF network collapse [7, 16, 17]. These collapses resemble aggregates of GFAP proteins, which occur when cells are transfected with mutant R416W GFAP [29]. This is one of the mutations in GFAP that causes Alexander disease (AxD), a fatal neurodegenerative disease characterized by leukodystrophy, macrocephaly, and psychomotor retardation [30]. A pathological hallmark of this disease is the presence of Rosenthal fibers, which are astrocytic aggregates that are comprised of GFAP, ubiquitinated proteins, and stress proteins, such as heat shock proteins like αB-crystallin (CRYAB) and heat shock protein 27 (HSP27) [29, 31–34], but also IF-associated proteins like plectin [35]. AxD mutations in GFAP and the subsequent collapse of the network influence astrocyte viability, morphology, and glutamate transport, and aggregates or accumulations of mutant AxD GFAP have a profound effect on astrocyte biology and physiology [36–39].

There is increasing evidence that GFAPδ changes IF properties [17, 40]. Here, we studied in more detail the differences in dynamic exchange of GFAPα and GFAPδ with the IF network in vitro. Furthermore, we analyzed the functional consequences of GFAPα or GFAPδ expression by assessing the effect of the altered GFAP network on the dynamics and localization of other IF proteins in the cells, on cell morphology, and focal adhesions. We also determined the effect on cell migration and proliferation, since it has been reported that GFAP affects these processes [4, 41–43]. Investigating the dynamic properties of the different GFAP isoforms and the effects of the different GFAP networks on cellular functions will contribute to our understanding of the consequences of modulation of the GFAP network for astrocyte physiology and pathology.

## Methods

### Cell culturing and transfections

U251MG human astrocytoma cells (gift from A.M.W. van Dam, VU University Medical Center, Department of Anatomy and Neurosciences, Amsterdam, The Netherlands) and U343MG human astrocytoma cells (gift from Prof Dr. R. Quinlan, Durham University, Durham, UK) were cultured in DMEM Glutamax (Gibco) mixed 1:1 with Ham’s F10 medium (Gibco) containing 10 % Fetal bovine serum (FBS) (Gibco) and 10-U/mL penicillin streptomycin (P/S) (Invitrogen). Human embryonic kidney (HEK293T) cells were cultured in DMEM/Glutamax with 10 % FBS, 1 % P/S, and 1 % extra Glutamax (all Invitrogen). All cells were cultured in uncoated plastic flasks (Corning) at 37 °C in a humidified atmosphere, with 5 % CO_2_.

### Isolation of primary human astrocytes

Primary human adult astrocytes were obtained from freshly dissected postmortem subcortical white matter of a 79-year-old female control donor (NBB 2010-038), with a postmortem delay of <18 hours (h) and a cerebrospinal fluid pH of 6.30. The tissue was obtained from the Netherlands Brain Bank (NBB), which performs brain autopsies with short postmortem intervals. The brain donors gave informed consent for using the tissue and for accessing the extensive neuropathological and clinical information for scientific research, which is in compliance with ethical and legal guidelines [44]. The tissue was collected in 25-mL cold Hibernate A (Invitrogen), and mechanically dissociated into small pieces. The tissue was digested with 0.2 % trypsin (Invitrogen) and 0.1 % DNAseI (Invitrogen) at 37 °C, while shaking for 30 min (min). Next, 2-mL FBS was added to the mixture, and subsequently, the cells were collected by centrifugation. The pellet was taken up in DMEM without phenol red containing 10 % FBS, 2.5 % Hepes, and 1 % P/S (all Invitrogen), and the suspension was filtered through a 60-μm mesh screen. Then, Percoll (Amersham/GE Healthcare) was added (half of the cell suspension volume), and this mixture was centrifuged at 3220 relative centrifugal force (rcf) at 4 °C for 30 min to separate cells, debris, and myelin. The second layer (glial cell containing fraction) was collected and washed with complete DMEM (containing 10 % FBS, 1 % P/S, 2.5 % Hepes, and 1 % gentamycin, all Invitrogen). After centrifugation, the pellet was taken up in complete DMEM, and cells were seeded in a 6-cm uncoated culture dish. Microglia adhered to the dish, and after 6 h at 37 °C/5 % CO_2_, the medium, containing astrocytes, was taken off, centrifuged, and the microglia depleted pellet was seeded onto poly-l-lysine-coated wells [PLL, Sigma-Aldrich, 15 μg/mL in PBS, 1 h at room temperature (RT)] in DMEM/Ham’s F12 GlutaMAX medium containing 5 % FBS, 1 % P/S, and 0.25 % Fungizone (all Invitrogen).

### Plasmid construction, transient transfection, and virus production

Expression vectors were prepared by cloning human GFAPα and human GFAPδ [7, 17, 29] full length cDNA sequences into the pIRES2EGFP (Clontech). For the GFAPδ constructs, the eGFP sequence was replaced by mCherry.

GFP tagged GFAP constructs were created by cloning human GFAPα and GFAPδ cDNA sequences [7] in frame after the eGFP sequence in peGFP using BAMHI and HindIII as restriction sites (Clontech) to create N-terminal eGFP tagged GFAPs. The N-terminal side was chosen for the eGFP tag, since GFAPα and GFAPδ differ in their C-terminal tail. All plasmids were sequenced.

Cells for fluorescence recovery after photobleaching (FRAP) experiments were transiently transfected using polyethylenimine (PEI) (Polysciences) or Lipofectamine (Invitrogen) according to manufacturer’s descriptions. 2.5 μg of plasmid DNA was used for PEI and 1.6 μg for Lipofectamine [5] in subconfluent 24-wells plates.

Subsequently, to produce lentiviral vectors, the constructs were subcloned into a pRRL lenti backbone. Lentiviruses were produced as described before [45, 46] with some alterations. In short, 10 × 10^6^ HEK 293T cells were plated in a 15-cm culture dish and transfected with a total of 90 μg of the envelope (pMD2.G), packaging (pCMV-dR8.74) and p156RRL plasmid, containing different expression cassettes per dish, using PEI. In total, 90 μg of DNA was mixed with PEI (67.5 ng/μL), incubated for 15 min at RT, and added dropwise to the cell culture. The culture medium was replaced 16 h after transfection, and the medium containing viral particles was collected 24 h after transfection. Supernatants were ultracentrifuged at 22,000 rpm (rotor SW28, Beckman-Coulter) for 2.5 h. The resulting pellet was resuspended in phosphate-buffered saline (PBS) (pH 7.4), aliquoted and stored at −80 °C until further use.

To measure viral titers, a dilution series across five orders of magnitude of the viral stock solutions was made and HEK293T cells were transduced. After 2 days of incubation at 37 °C, the number of transduced fluorescent cells at the different viral dilutions was counted, and the viral titer was determined in transducing units (TU)/mL.

### Creating stable cell lines

For functional experiments, cell lines expressing GFAP isforms were created. U251 cells were transduced with lentiviral constructs with a multiplicity of infection of 10. Medium was refreshed after 16 h. To maintain a population of transduced cells, cells were sorted on their EGFP or mCherry expression using fluorescent activated cell sorting (FACS ARIA II, BD Bioscience, Franklin Lakes, NJ, USA). In between experiments, U251 cells were stained for GFAP to ensure that more than 70 % of the cells were expressing the construct. The primary human astrocytes were checked for fluorescent reporter expression before any analysis to ensure cells were expressing GFAP isoforms.

### MTT assay

To measure cell proliferation, an MTT(3‐(4,5‐Dimethylthiazol‐2‐yl)‐2,5‐diphenyltetrazolium bromide) assay was performed. MTT is reduced into a soluble blue formazan product by mitochondrial enzymes in living cells only. Therefore, the amount of formed formazan is proportional to the amount of living cells present [47]. MTT assays were performed by plating cells in non-coated plastic 24-wells plates (Greiner). To quantify cells, medium was replaced by 500-μL serum free medium containing 0.5-mg/mL MTT, which was incubated at 37 °C for 2 h. Cells were subsequently lysed in 100 % DMSO, which dissolves the purple formazan resulting in a color change of the DMSO. The amount of purple formazan, and therefore the amount of cells able to metabolize the MTT, was measured using a Varioskan Flash (Thermo scientific, USA), measuring the absorbance at 570 nm. Significance was tested with a Kruskal–Wallis test with Dunn’s Multiple Comparison post hoc test on 3 independent experiments. Every measurement in the independent experiments was the average of a biological duplicate.

### Phospho-histone H3 quantification

To determine the number of actively proliferating cells, U251 cells expressing GFAP isoforms were plated on non-coated coverslips and fluorescently stained for phospho-histone H3 (PHH3) together with the nuclear dye Hoechst (1:1000 dilution) (Invitrogen). Subsequently, micrographs were taken, the number of PHH3 positive, dividing, nuclei was counted using ImagePro software (version 6.3), and the percentage of dividing cells was calculated by dividing the number of PHH3 positive cells by total number of Hoechst positive nuclei. Per experiment, 5 fields of view were analyzed and averaged, each containing at least 50 cells. Data from separate experiments were corrected for inter-experimental variation as stated below. Significance was tested with a Kruskal–Wallis test with Dunn’s post hoc test on data from 3 independent experiments.

### Migration assay

To measure cell migration, a scratch assay was performed. U251 cells were plated in a 24-wells plate (100,000 cells per well) coated with 20-μg/mL PLL at 37 °C for 1 h. The confluent cell monolayer was scratched with a P10 plastic pipet tip. Pictures were taken using an Axiovert 135 M (Zeiss) with a Sony XCD-X700 camera (Sony) at the time points indicated in the results. To quantify cell migration, the surface area not covered by cells was determined at different time points. The migration was calculated as the percentage of uncovered surface area compared with *t* = 0. A mean of 9 pictures was measured per condition, in at least 3 separate experiments. Significance was tested with a Kruskal–Wallis test with Dunn’s post hoc test.

### Single cell motility assay

Single cell motility assays were performed on a Zeiss Axiovert 2000 inverted microscope (Zeiss, Jena, Germany). A single cell suspension was plated on PLL-coated glass dishes with four compartments (CELLview, Greiner bio-one, Alphen a/d Rijn, The Netherlands) and allowed to adhere for at least 8 h. Dishes were kept on the microscope in a pre-heated and humidified incubation chamber (OKO labs) at 37 °C and 5 % CO_2_. Pictures were taken every 10 min with an Axi Aqua camera (Q imaging). Cell motility was measured by tracking single cells throughout all frames of the sequence and measuring the average velocity in μm per min using the manual tracking plugin from ImageJ (Rasband, W.S., ImageJ, U. S. National Institutes of Health, Bethesda, Maryland, USA, http://imagej.nih.gov/ij/, 1997–2012 version 1.46f). Per experiment, at least 20 cells were analyzed per condition in at least 3 independent experiments. Data from separate experiments were corrected for inter-experimental variation as stated in the “Statistics and factor correction” section below. A Kruskal–Wallis test with Dunn’s post hoc test was performed to test for significance. For the primary human astrocytes, which were not sorted, we checked for GFP and mCherry expression to make sure that we only tracked transduced cells.

### Quantitative reverse transcriptase PCR (qPCR) analysis

U251 cells were transduced like described before. Medium was refreshed after 16 h. RNA was extracted 7 days after transduction. RNA was extracted from cells using TRIsure (Bioline, London, UK) and precipitated in isopropanol overnight (O/N). Five hundred nanograms of RNA was reverse transcribed into cDNA with a QuantiTect reverse transcription kit (Qiagen), as described before [5]. cDNA was diluted 1:20 before being used as a template in qPCR assays (SYBR^®^ Green PCR Master Mix, Applied Biosystems). qPCR conditions were similar as described before [5] and glyceraldehyde-3-phosphate dehydrogenase (GAPDH) and hypoxanthine phosphoribosyltransferase (HPRT) were used as reference genes to normalize gene expression. Data from 4 separate experiments were factor corrected as stated in the “Statistics and factor correction” section below and tested for significance using a Kruskal–Wallis test with Dunn’s post hoc test. Primer pairs used are listed in Table 1.

**Table 1:** qPCR primer pairs of human GFAP isoforms

Transcript	Forward primer	Reverse primer
*GFAPα endogenous*	CCCACTCTGCTTTGACTGAGC	CCTTCTTCGGCCTTAGAGGG
*GFAPδ endogenous*	GTGGTAAAGGTGGTGAGTCCTT	AGAGGCTGCTGCTTGCTC
*Vimentin*	CGTACGTCAGCAATATGAAAGTGTG	TCAGAGAGGTCAGCAAACTTGGA
*Nestin*	GATCTAAACAGGAAGGAAATCCAGG	TCTAGTGTCTCATGGCTCTGGTTTT
*GFAPα*	CTTCTCCAACCTGCAGATTCG	CACGGTCTTCACCACGATGTT
*GFAPδ*	CCGTGCAGACCTTCTCCAA	CGTATTGTGAGGCTTTTGAGATATCT
*GFAPκ*	GTCAGTACAGCAGGGCCTCG	AGGAGCGCTGCAGTGTCACG
*GFAPβ*	CGGGCATCGCCAGTCTAG	ATCCTGCTCTGGCTCTGCTC
*GFAPγ*	CTCAGAAGAGCCTGGACCCA	GGCTTCCAGCCTCAGGTTG
*GFAPζ*	GCACTGTGCACGTTCCCTG	GGTCCTGCCTCACATCACATC
*GFAPΔEx6*	TGCGCGGCACGGATC	CACGGTCTTCACCACGATGTT
*GFAPΔ135*	TCTGCGCGGCACGGAGTA	GGGAATGGTGATCCGGTTCT
*GFAPΔ164*	GAGGCGGCCAGTTATTCCC	CACGGTCTTCACCACGATGTT
*GFAPΔEx7*	GCGAGGAGAACCGAAACCAG	CTTCACCACGATGTTCCTCTTG

Endogenous GFAPα and GFAPδ primers are located in the 3′UTR part, which is absent in the plasmid cDNA. The primer sequences used were published before [15]

*GFAP* glial fibrillary acidic protein

### Western blots

Cells were washed and collected with a cell scraper into 100 µL of cold lysis buffer consisting of a suspension buffer (0.1 M NaCl, 0.01 M Tris–Hcl (pH 7.6), 1-mM Ethylenediaminetetraacetic acid (EDTA) with 1 % Triton-x100) and protease inhibitors were added (100-μg/mL phenylmethanesulfonylfluoride (PMSF)(Roche Diagnostics) and 0.5-μg/mL Leupeptin (Roche Diagnostics). Cells were vortexed and incubated for 5 min on ice. Subsequently, samples were spun at 11.7 k rcf for 1 min. Supernatant was taken off and stored at −20 °C until further use. Protein concentrations were measured using a BCA kit (Pierce, Thermo Scientific), according to manufacturers descriptions. Proteins were mixed with 2X loading buffer (2X: 100 mM Tris pH 6.8, 4 % SDS, 20 % glycerol, 0.2 M dithiothreitol, and bromophenol blue), heated for 5 min at 95 °C, and loaded on a 7.5 % SDS-PAGE reducing gel. After electrophoresis, proteins were blotted on Whatman Protran membranes (GE Healthcare) using a semi-dry Trans-Blot system (Biorad) for 60 min. Blots were incubated with SuMi (50 mM Tris, 150 mM NaCl, 0.25 % gelatine and 0.5 % Triton X-100, pH 7.4) for 10 min before they were incubated with primary antibodies at 4 °C overnight. Blots were subsequently washed 3 times in TBS-T (100 mM Tris–Hcl pH 7.4, 150 mM NaCl with 0.2 % Tween-20), before secondary antibodies [IRdye 800 (1:2000) (LI-COR) and Dyelight Cy5 (1:4000) (Jackson Immune Research), diluted in SuMi were incubated at room temperature for 1 h. Blots were washed again 3 times in TBS-T before scanning with an Odyssey scanner (LI-COR). GAPDH was used as a loading control.

### Immunocytochemistry

To perform immunocytochemical staining, cells were cultured on uncoated glass coverslips, fixed with 4 % Paraformaldehyde (PFA), washed in PBS, and incubated in SuMi buffer for 10 min. Primary antibodies were diluted in SuMi and incubated at 4 °C on a shaker O/N. Cells were washed 3 times in PBS and, subsequently, incubated with secondary antibodies and Hoechst 33258 (1:1000 dilution) (Invitrogen) diluted in SuMi at RT for 1 h. The antibodies used are listed in Table 2. All secondary antibodies were from Jackson Immune Research and diluted 1:1400 in SuMi. Cells were washed again in PBS, before the coverslips with cells were mounted on slides with Mowiol [0.1 M Tris–HCl pH 8.5, 25 % glycerol, 10 % Mowiol (Calbiochem, Merck Millipore)]. The actin network was visualized with acti-stain Phalloidin 670 (Cytoskeleton inc; 1:1000 dilution). All fluorescent images were taken with a Leica SP5 confocal microscope (Leica) with a 63x objective.

**Table 2:** Primary antibodies

Antibody	Manufacturer	Dilution	Cat #
Pan GFAP Dako	Dako	1:4000 (1:8000 WB)	Z0334
hGFAPδ	Manufactured in house (10-05-2001 Bleed)	1:1000 (1:1300 WB)	–
GFAP c-term	Santa Cruz	1:4000 (WB)	Sc-6170
Vimentin	Chemicon	1:3000	AB5733
GAPDH	Abcam	1:4000 (WB)	AB14247

*GFAP* glial fibrillary acidic protein, *WB* western blot, *c-term* carboxy terminal, *hGFAP* human GFAP, GAPDH glyceraldehyde-3-phosphate dehydrogenase

### Cell morphology measurements

Phase contrast pictures were taken from the U251 cells transduced with GFAP isoforms and mCherry (control) on a Zeiss Axiovert 2000 with an Exi Aqua camera (Q Imaging, Surrey, Canada). Cell outlines were manually drawn using Image J. Area and perimeter were measured in square units. Form factor was calculated as $4 \pi \frac{({\rm Area})}{({\rm Perimeter})^2}$, where perfectly round cells will have a form factor of 1 [43, 48]. Five independent experiments were performed with 40 cells analyzed per experiment. Data have been factor corrected for inter-experimental variation as stated below. A Kruskal–Wallis test was performed with Dunn’s post hoc test to test for significance.

### Live cell imaging

U343MG cells were imaged for 48 h using a Leica IR-BE (Leica Microsystems GmbH) inverted wide field microscope at 37 °C in a custom built incubator containing 5 % CO_2_. Phase contrast and fluorescence images were acquired with a 40x objective at 10 and 30 min time intervals during 48 h. The single images were reconstructed and rendered into a time-lapse using Huygens software (Scientific Volume Imaging) and Image Pro Plus (Mediacybernetics).

### Fluorescent recovery after photobleaching (FRAP)

FRAP analysis was performed on transiently transfected U251MG cells. During imaging the temperature was maintained at 37 °C in a humidified incubator chamber (OKO labs). Cells were analyzed 24 h after transfection. To monitor dynamics of GFAP in collapsed networks, we transfected the cells with GFAPδ in combination with either GFP–GFAPα or GFP–GFAPδ. FRAP experiments were carried out on a SP5 Leica Confocal Microscope (Leica) with a 63x objective. The pinhole was set on 209.99 µm, and the scanning speed was at 400 Hz with a resolution of 512 × 512 pixels. Bar-shaped regions of interest (ROI) of 1.5 µm × 10 µm were bleached with a 488-nm Argon laser (full laser power) until at least 50 % of the fluorescence was bleached. Immediately after bleaching, a time-series of capturing 10 frames with a 30 s interval were made. Then, z-stacks were taken manually every 5 min up to 30 min after bleaching. To ensure that the bleached ROI did not drift out of focus, z-stacks were made throughout the whole cell. Three ROIs were bleached per cell at different locations within the IF network. FRAP experiments were performed at different days in at least three separate experiments. This resulted in the following amount of ROIs measured: 33 × GFAPα in a network, 30 × GFAPδ in a network, 8 × GFAPα in a collapse, and 8 × GFAPδ in a collapse.

### FRAP analysis

ROIs were positioned in post bleach pictures manually. Per time point, the position of the ROI was corrected for cell movement, and average fluorescence was measured using Image J. The average fluorescence was plotted in time to obtain fluorescence recovery curves for every ROI. The half time was calculated by interpolating the time at 50 % of the fluorescence of the maximum fluorescence at 30 min. The immobile fraction was calculated by comparing the fluorescence in the bleached area after recovery (*F*_∞_) with the fluorescence before bleaching (*F*_i_) and just after bleaching (*F*_0_). The immobile percentage is defined as (1 − (*F*_∞_ − *F*_0_)/(*F*_i_ − *F*_0_)) × 100. For *F*_∞_, the average of the last three time points was used for analysis.

### Statistics and factor correction

Data obtained from independent experiments were corrected with a factor correction program (version 10.5 2012) [49] when stated. Kruskal–Wallis, Kolmogorov–Smirnov, or Mann–Whitney tests were performed to test for significance. Differences were considered significant at *p* < 0.05. All statistical tests were performed using Graphpad Prism 5 (version 5.04) (Graphpad Software Inc., La Jolla, CA, USA).

### Focal adhesion analysis

To analyze focal adhesions, coverslips were coated with laminin in 24-wells plates. Cells were plated at 10,000 cells per well. After attaching for 2 days, cells were fixed with 4 % PFA, stained with rabbit anti-phosphorylated-paxillin (pY118; Life Technologies), and counterstained with Hoechst and Phalloidin-488.

Fixed and immunostained samples were imaged on an inverted microscope (Zeiss Axiovert 200) with a Confocal Spinning-disk Unit (Yokogawa CSU X-1) and an emCCD camera (Andor iXon 897). Excitation was accurately controlled by 405 nm (Crystalasers), 514 nm (Cobolt), and 642 nm (Spectra Physics) lasers through an acousto-optic tunable filter (AA Optoelectronics) coupled into the CSU with a polarization-maintaining optical fiber. Images were acquired with Andor IQ 2 software, and further processing and analysis were performed in specifically designed software (Matlab, Mathworks).

Focal adhesions were automatically detected from the 642-nm fluorescence images. Focal adhesion size could be at least 0.4 × 0.4 μm and at most 4 × 4 μm. In an image of 512 × 512 pixels low pass frequency with a cut-off at 2 pixels filtering removed large features. With a subsequent threshold of 3 times the standard deviation of the image, we obtained and characterized the focal adhesions. Focal adhesion size differences were tested using a 2-tailed Kolmogorov–Smirnov test. Differences were considered significant if *p* < 0.05. Per condition, a different amount of cells and focal adhesion were measured, but at least exceeded 30 different cells and a total of more than 700 focal adhesions.

## Results

### GFAPδ perturbs the GFAP network in astrocytes

Cellular models for studying GFAP isoform function were established by expression of GFAP isoforms in U251 cells and primary human astrocytes. The mRNAs of the different isoforms were highly expressed in the transduced U251 cell lines (Sup. Fig. 1a, b) and primary human astrocytes (Sup. Fig. 1d, e). Western blot analysis showed a clear expression of either GFAPα or δ (Sup. Fig. 1c), while the control (Ctrl) only showed a band with the pan GFAP antibody, representing endogenous GFAPα. The endogenous expression of the other GFAP isoforms was determined with qPCR and is presented as a percentage of the canonical GFAPα expression. GFAPα and GFAPδ were the most abundant isoforms expressed, followed by GFAPκ. Other isoforms were expressed at a very low level (Sup. Fig. 1f).

**Fig. 1:**
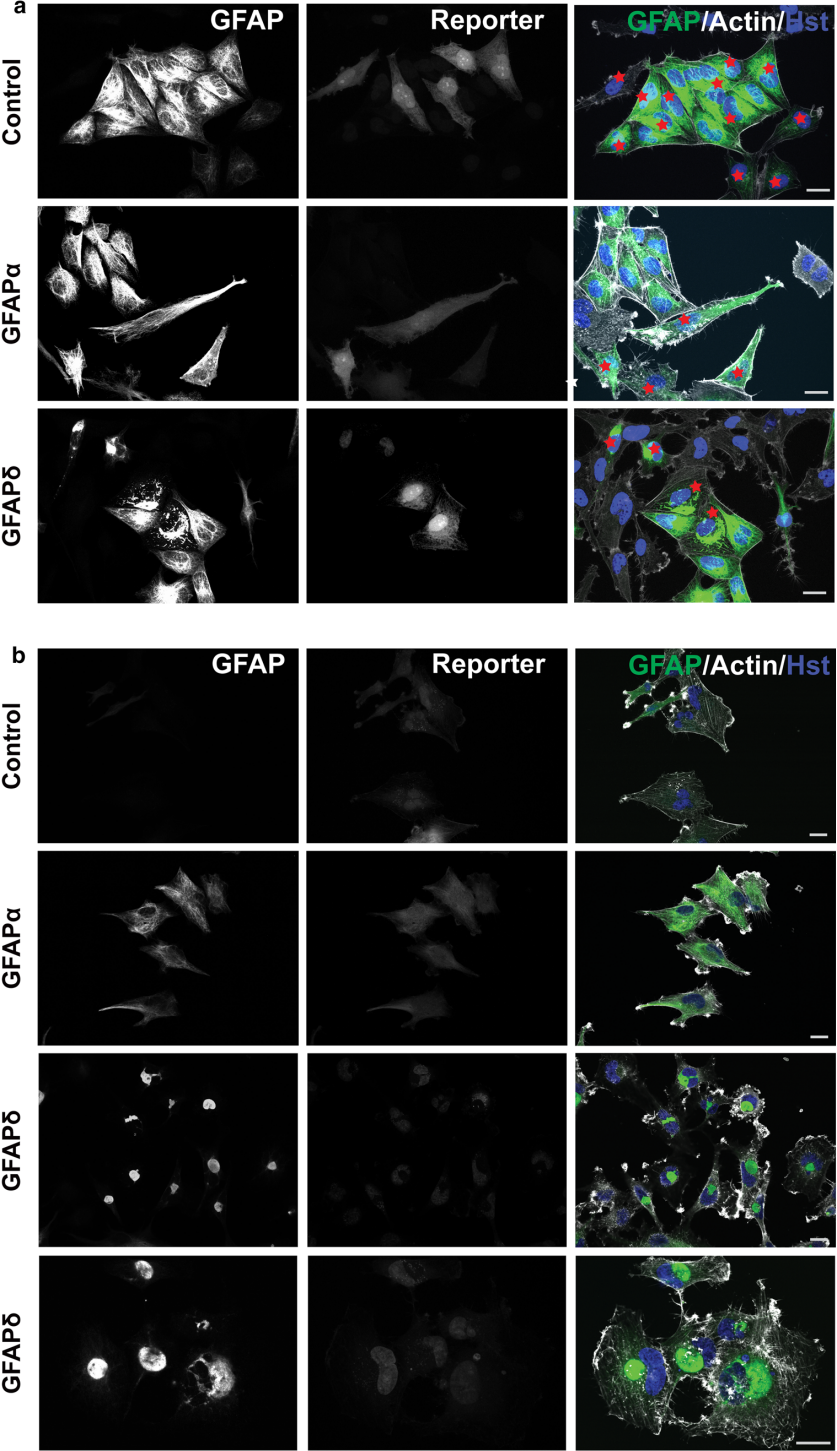
GFAP isoform expression in primary human astrocytes and U251 astrocytoma cells. **a** Human primary astrocytes transduced with GFAPα, GFAPδ, or mCherry (control). Cells were stained with pan GFAP Dako antibody (GFAP) together with phalloidin (Actin) and Hoechst (Hst). The reporter showed in the *middle panel* which cells were transduced cells, and these are additionally highlighted in the *right panel* with *red stars*. Expression of GFAPα resulted in a dense GFAP network, which was spread throughout the whole cell. Expression of GFAPδ showed a drastic redistribution of the GFAP network, which collapsed in a perinuclear fashion. **b** Human U251 astrocytoma cells transduced with GFAPα, GFAPδ, or mCherry (control) also showed a collapse of the GFAP network in GFAPδ transduced cells only. In this panel, all cells were transduced, as these were stably transfected cell lines. A close up of the collapsed network is shown in the *lowest panel*. *Scale bars* represent 20 μm

First, we confirmed whether GFAPδ-induced cytoplasmic collapses of GFAP also occurred in the transduced U251 cells and primary human astrocytes, as we have shown before in SW13 human adrenal carcinoma cells and U343 astrocytoma cells [5, 17]. We investigated the IF network morphology by immunostainings for GFAP, and we observed that the different GFAP isoforms gave similar results in both the primary human astrocytes (Fig. 1a) and the human U251 astrocytoma cells (Fig. 1b). The endogenous GFAP network was stained in the control condition, in which cells were transduced with mCherry. The GFAP network was present throughout the cytoplasm up to close proximity of the cell periphery, which is visualized by actin staining. Expression of recombinant GFAPα in human astrocytes, or in U251 cells, resulted in a GFAP network which was morphologically similar to astrocytic IF networks, indicating that the recombinant GFAPα was incorporated into the endogenous IF network of the cells. In contrast, expression of GFAPδ led to a collapse of the GFAP network mostly in a perinuclear fashion (Fig. 1a, b). This perturbing effect of GFAPδ on the GFAP network in these cells is concentration dependent and is a gradual process (data not shown), as has also been described before [16, 17].

### Vimentin and nestin co-collapse with GFAPδ, while actin and microtubules stay intact

**Fig. 2:**
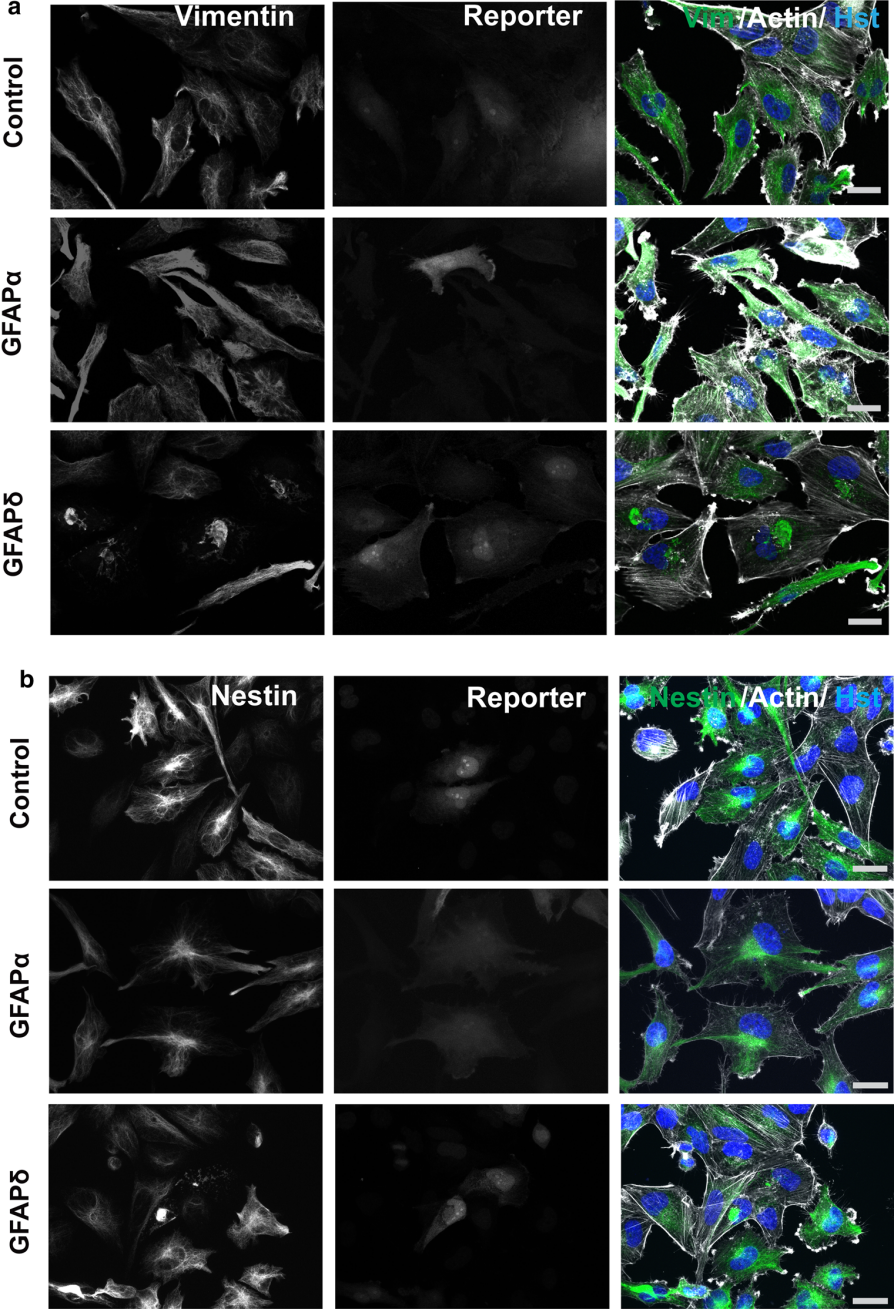
GFAPδ collapses the whole IF network. **a** Primary human astrocytes transduced with GFAPα, GFAPδ, or control plasmid stained for vimentin and actin or **b** nestin and actin. Astrocytes expressing ectopic GFAPα or mCherry had a network that was spread throughout the whole cell while GFAPδ expressing cells showed a perinuclear collapse of both vimentin and nestin in cells positive for the reporter only. GFAPδ transduced cells with relative low expression, as seen by lower reporter expression, do not show a collapsed IF network yet. *Scale bar* represents 20 μm

To assess whether GFAPδ induces a collapse of the complete cytoplasmic IF network, we also studied the distribution of vimentin (Fig. 2a) and nestin (Fig. 2b), which were both present in the IF network in primary human astrocytes. Transduction with GFAPα had no effect on the intracellular location of these proteins, but transduction with GFAPδ led to a condensation of vimentin and nestin, mostly around the nucleus. The same effect was observed in the U251 cell line (not shown). The actin and microtubule cytoskeletal networks did not co-collapse with the IF network upon GFAPδ expression (Fig. 2a, b, Sup. Fig. 2a, b). The staining intensity of actin was very variable between cells and between conditions, but the overall morphology of the actin network showed no actin collapse induced by GFAPδ.

To examine whether the additional expression of GFAPα or GFAPδ would lead to effects on transcription of other IFs, we analyzed the change in mRNA expression of vimentin, nestin, and endogenous GFAPα and GFAPδ in U251 cells using quantitative PCR. GFAPα mRNA (Sup. Fig. 3a), GFAPδ mRNA (Sup. Fig. 3b), vimentin mRNA (Sup. Fig. 3d), and vimentin protein (Sup. Fig. 3e) levels were not significantly changed due to an increase in either GFAPα or GFAPδ. However, we noticed a small but significant upregulation of nestin transcript in GFAPα expressing cells (*p* = 0.03) (Sup. Fig. 3c).

### GFP tagged GFAP incorporates into the endogenous IF network

Next, we assessed the dynamic properties of GFAP isoforms using N-terminally GFP tagged GFAP fusion proteins for live cell imaging. The transduced cells express GFP-labeled GFAP isoforms, and thereby, transduced cells can be directly identified based on the reporter GFP. Both GFP–GFAPα and GFP–GFAPδ did incorporate into the endogenous IF network of U251 cells (Sup. Fig. 4a, b). Since a high expression of tagged GFAP also led to a collapse of the network, a relatively low expression was needed to image a non-collapsed network, which was established by analysis 24 h after transient transfection using U251 cells for a reasonable transfection efficiency. 24 h after transfection, the GFP–GFAPα transfected cells showed a spread out network (Sup. Fig. 5a), and GFP–GFAPδ expressing cells showed a mixture of cells with either a spread out network (Sup. Fig. 5b) or a collapsed IF network (Sup. Fig. 5c). The endogenous IF network is visualized by the vimentin staining. About 30 % of the GFP–GFAPδ expressing cells in our cell culture condition showed a collapse at this time point, although the exact percentage varied between experiments and was dependent on the transfection efficiency.

### Live cell imaging of the GFAPδ dynamically shows the collapse of the network

**Fig. 3:**
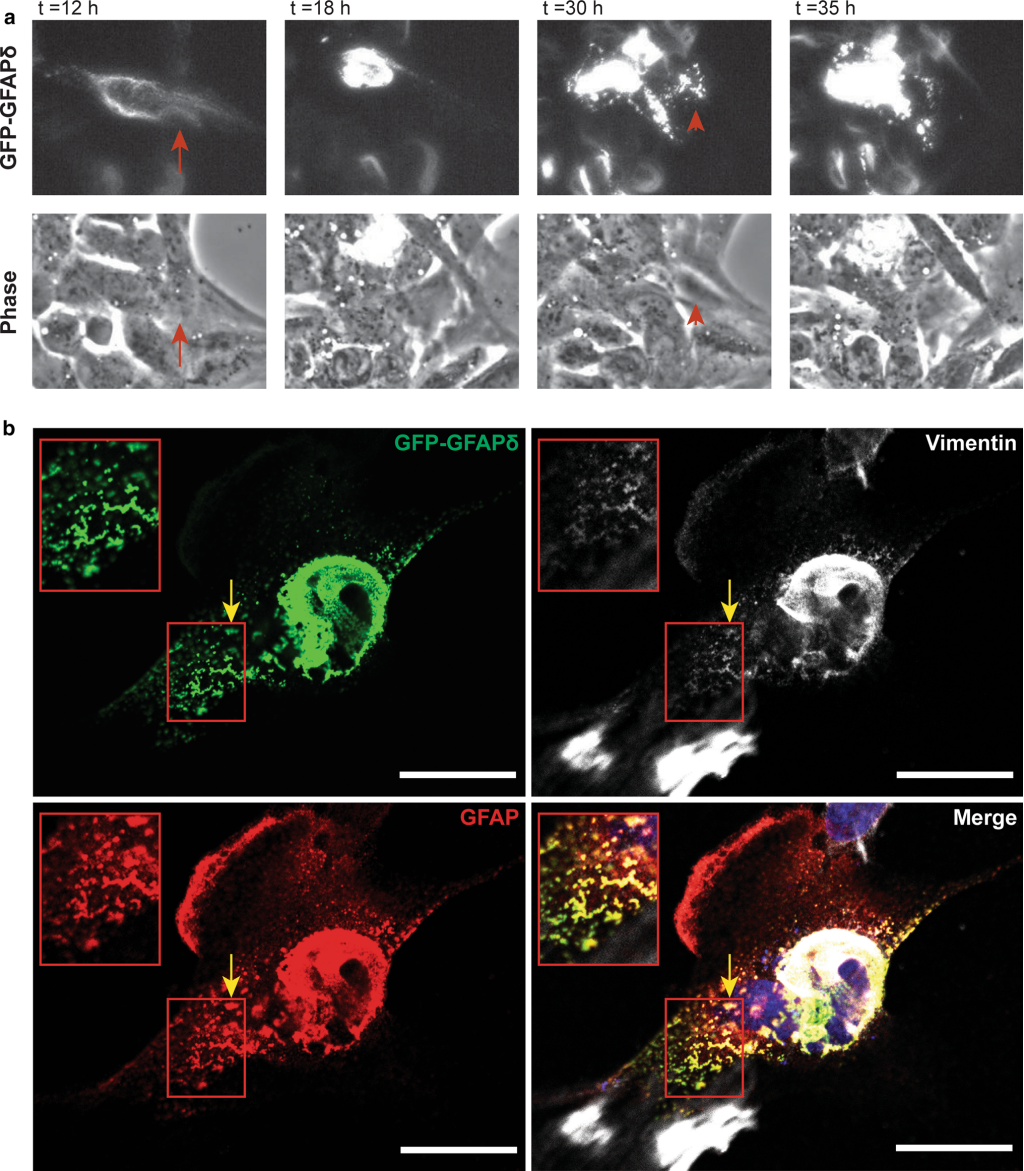
Collapse of the IF network due to high GFP–GFAPδ expression. **a** Stills from a representative live cell imaging experiment. U343MG cells were transfected with GFP–GFAPδ and imaged for 48 h. The GFP–GFAPδ was initially incorporated into the IF network (*arrows* at *t* = 12 h), but as the amount of GFP–GFAPδ increased over time, it eventually caused a collapse of the network (*t* = 18 h). During the process of collapsing, thicker and shorter filamentous structures are visible in the cell, which are moving into the direction of the collapsed network (*arrowheads* in *t* = 30 h). **b** These small filaments sometimes co-localized with vimentin in U251MG cells as well (*yellow arrow*). *Scale bars* represent 20 μm. See also supplemental Movie 1 and 2

To visualize the dynamics of the collapsing network over time, U343MG astrocytoma cells transfected with GFP–GFAPδ were imaged for 48 h, starting 4 h after transfection. U343MG cells were used here, since they are less motile than U251MG cells, which enabled us to image the IF network for a long period of time. First, GFP–GFAPδ was distributed throughout the IF network in the whole cell (arrow in Fig. 3a *t* = 12 h). GFP–GFAPδ started to condensate around the nucleus, as the expression of GFP–GFAPδ increased over time (Fig. 3a and Sup. Movies 1 and 2). Sometimes small aggregates or condensations were seen, which joined the already collapsed GFAP proteins (arrowheads Fig. 3a *t* = 30 and Sup. Movies 1 and 2). During and after the process of the accumulation, cells were still migrating, and the collapsed IF network was a motile structure in both stationary and moving cells. These experiments showed that GFP–GFAPδ, in small amounts, is incorporated into the endogenous IF network before it causes a collapse of the IF network. Images from fixed cells stained for GFP, GFAP, and vimentin showed that the squiggles (short filaments) of GFP–GFAPδ sometimes co-localized with vimentin and were not part of larger filaments (Fig. 3b, arrow).

### Dynamic properties of GFP–GFAPα are different from GFP–GFAPδ

**Fig. 4:**
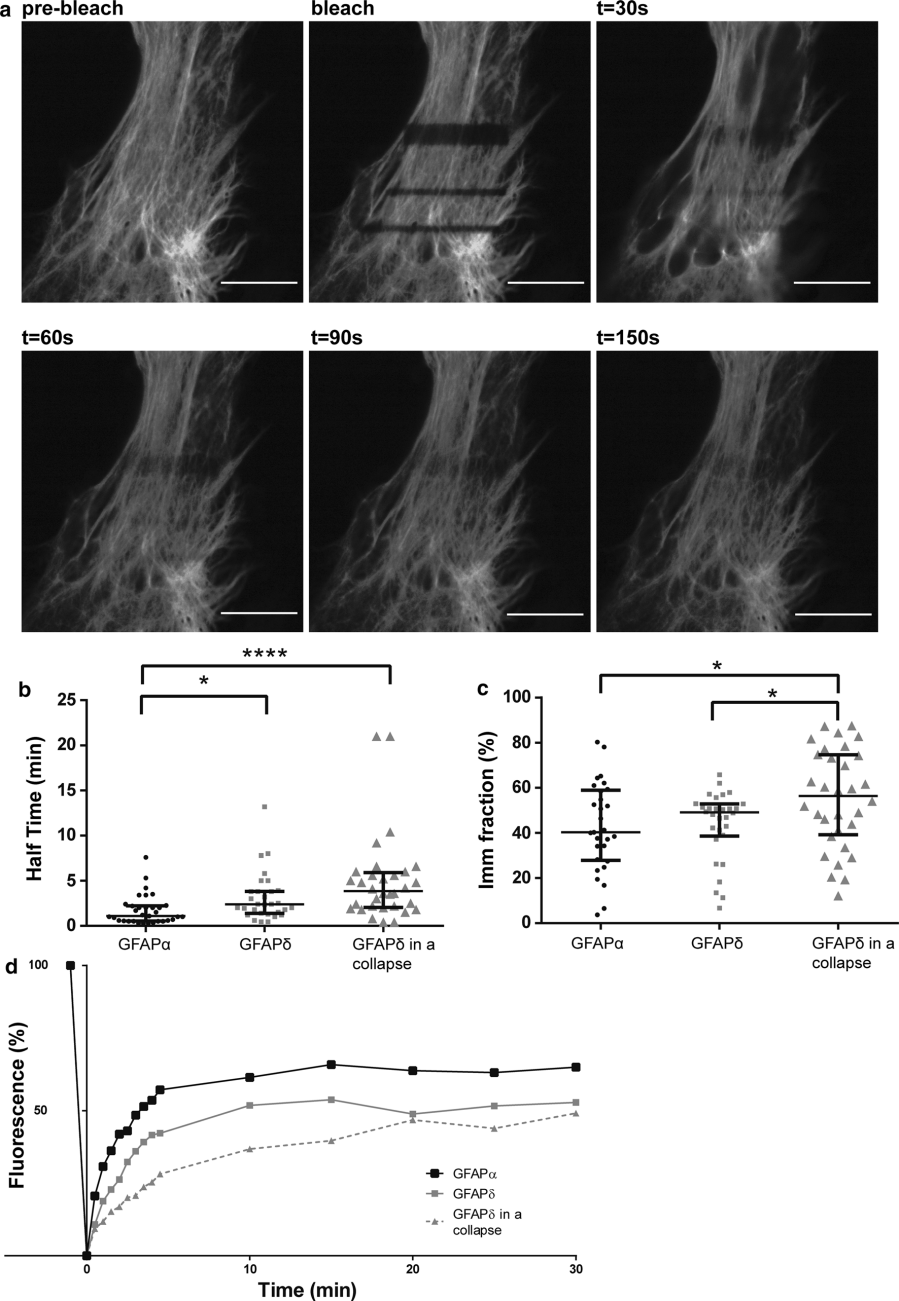
Different dynamics between GFAPα and GFAPδ. **a** FRAP experiments consisted of bleaching ROI and measuring the fluorescence recovery up to 30 min after the bleach in U251MG cells. Within 150 s, most of the fluorescence was recovered, but recovery was never complete. **b** FRAP experiments were performed for GFAPα, GFAPδ, and GFAPδ in a collapsed network, and half times were calculated. There is a significant difference in half time between GFAPα (median = 1.1 min) and GFAPδ (median = 2.3 min) (*p* < 0.05; *n* = 33, *n* = 30), and between GFAPα and GFAPδ in a collapsed network (median = 3.8 min) (*p* = 0.000; *n* = 32). There was no significant difference in the half time of GFAPδ in spread out or collapsed network, although there was a trend that a collapse decreased the half time of GFAPδ. **c** The immobile fractions were not significantly different between GFAPα (40.2 %), GFAPδ (49.1 %) (*p* = 0.7; *n* = 29 and *n* = 30). GFAPδ in a collapse did have a significantly different immobile fraction (56.3 %; *n* = 32) compared with GFAPα (*p* = 0.03) and GFAPδ (*p* = 0.04) in a network. Graphs** b** and** c** show median values with interquartile range. **d** Medians of FRAP curves for GFAPα, GFAPδ and GFAPδ in a collapse show the recovery after bleaching. Non-parametric tests were performed on the data extracted from the FRAP measurements, so non-overlapping curves do not equal significant differences in this graph

To assess the dynamic properties of GFAPα and GFAPδ, FRAP experiments were performed on U251 astrocytoma cells in which small boxed regions of fluorescent cells were photobleached, and recovery of fluorescence was measured over time. Cells were transfected with the GFP–GFAP isoforms, and the FRAP analysis was started 24 h later. Cells with non-collapsed and collapsed networks were measured and analyzed separately. Regions of fluorescent GFAP networks were bleached, and the fluorescence recovery was imaged up to 30 min after bleaching. A typical example is shown in Fig. 4a. The median of all FRAP recovery experiments for GFAPα and GFAPδ in an extended network and GFAPδ in a collapsed network are shown in Fig. 4d. The half time *t*_½_ (the time needed to recover to 50 % of the final fluorescence) and the immobile fraction (the percentage of fluorescence which is not recovered) were calculated. The median *t*_½_ value of GFAPδ in extended, non-collapsed networks was 2.3 min, and this was significantly (*p* < 0.05) longer than the median *t*_½_ of GFAPα, which was 1.1 min (Fig. 4b). Since high GFAPδ expression leads to a collapse of the IF network, we next investigated the dynamic properties of GFAPδ in a collapsed network. Although not significant, there was a clear trend visible that GFAPδ had a longer *t*½ (median = 3.8 min) when in a collapsed network compared with GFAPδ in a spread out network (Fig. 4b), indicating a slower on/off rate from the IF network. We also calculated the immobile fraction of the GFP–GFAPs, which is the percentage of fluorescence that did not recover from the FRAP curves. We observed that the immobile fraction did not significantly differ between GFAPα (median = 40.3 %) or GFAPδ in extended network (median = 49.1 %). There was, however, a significant difference between the immobile fraction of GFAPδ when the majority of the GFAP was in a collapsed network (median = 56.4 %) compared with the immobile fraction of GFAPα and GFAPδ when GFAP was in a spread out network (Fig. 4c).

**Fig. 5:**
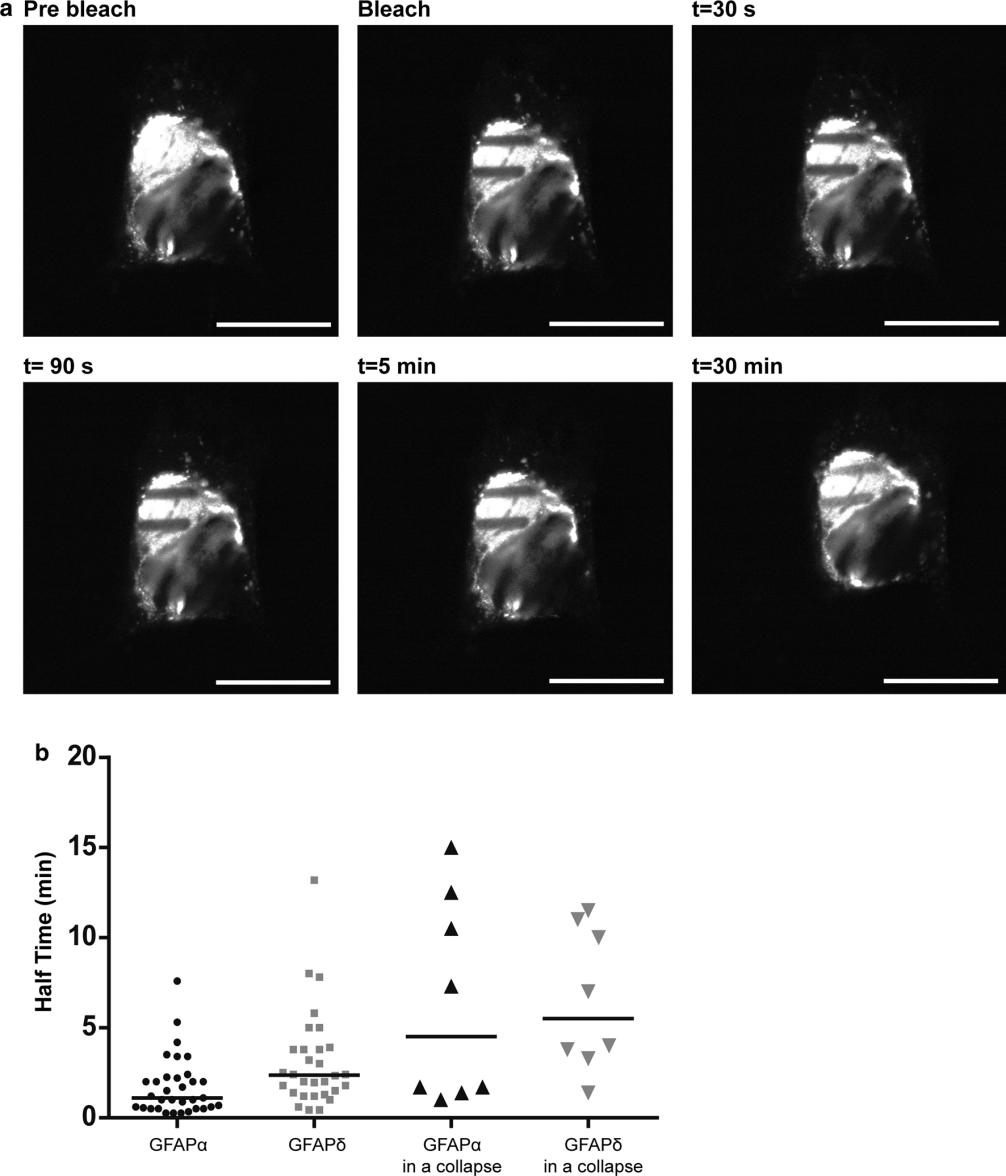
Dynamics of GFAP isoforms in a collapsed IF network. **a** A representative still of a FRAP experiment on U251MG cells with a collapsed IF network. ROIs were bleached and the fluorescence recovery was measured for up to 35 min. Even after 30 min, the bleach area was still clearly visible. **b** Half times were calculated as described in the materials and methods section. There were no significant differences between the half time of GFAPα in a collapse (median = 4.5 min) or GFAPδ in a collapse (median = 5.5 min) (*p* = 0.8; *n* = 8). There was a clear trend showing that a collapse caused a longer half time (4.5 vs. 1.1 min for GFAPα and 5.5 vs. 2.3 min for GFAPδ) of the GFAP isoforms

To assess whether the change in dynamic properties between GFAPδ when in a network and when it has collapsed was due to the collapse of the network and not GFAPδ itself, we performed experiments in which we also measured the dynamics of GFAPα in a collapsed network. To study this, we transfected cells with untagged GFAPδ to induce the collapse, and co-transfected either GFP–GFAPα or GFP–GFAPδ to visualize the fluorescent isoform dynamics. Both GFP–GFAPα (Sup. Fig. 5a) and GFP–GFAPδ (Sup. Fig. 5b) were incorporated into the collapse as can be seen by staining for vimentin, which is highly expressed in U251 cells and shows the endogenous IF network. A representative example of a bleached collapsed network and the subsequent recovery is shown in Fig. 5a. The median *t*_½_ for GFAPα (4.4 min) and GFAPδ (5.5 min) did not differ significantly (*p* = 0.8) (Fig. 5b). The *t*_½_ measured for GFAPα was remarkably increased due to the collapsed network (Fig. 5b). The fluorescence had not yet reached plateau after 30 min of recovery. Taken together, this indicates that GFAPα and GFAPδ have different exchange dynamics (as measured by *t*_½_) and the dynamics show a trend for higher *t*_½_ when the IF network is collapsed.

### A collapsed IF network changes cell morphology

**Fig. 6:**
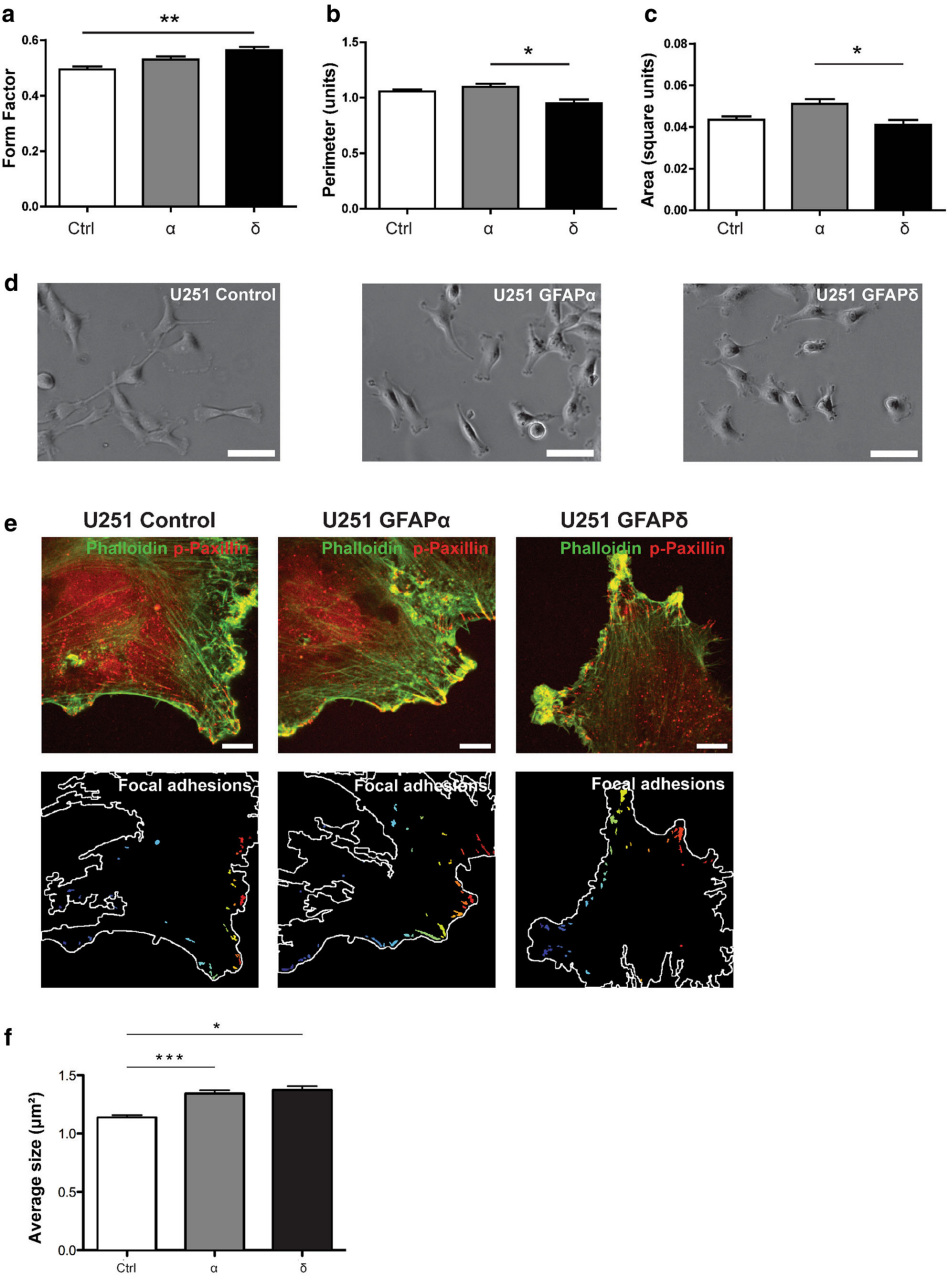
Morphology of U251 cells with different IF networks. **a** GFAPδ expressing cells showed a more round morphology in comparison to the control (*p* = 0.009) as is measured by the form factor of these cells. Significant differences between GFAPα and GFAPδ were found in **b** perimeter (*p* = 0.02) and **c** area (*p* = 0.03). *Bars* show mean and SEM (*n* = 5). **d** Phase contrast pictures showing the more round morphology of the GFAPδ expressing cells in comparison with the control vector and GFAPα. *Scale bars* represent 100 μm. **e** U251 cells expressing different GFAP isoforms are stained for actin with phalloidin (*green*) and phosphorylated paxillin (*red*). *Scale bars* represent 10 µm. The *lower panel* shows focal adhesions (overlap of phalloidin and phosphorylated paxillin). **f** Quantification of focal adhesion shows larger focal adhesion size in GFAPα (*p* = 0.0001) and GFAPδ (*p* = 0.001) expressing cells compared with the control mCherry (mCherry: 1.13 μm^2^ ± 0.02 *n* = 735; GFAPα: 1.34 μm^2^ ± 0.03 *n* = 994; GFAPδ: 1.37 μm^2^ ± 0.03 *n* = 828

Next, we aimed at defining whether the IF network collapse and changes in dynamics also result in a change in morphology, since IF expression has been linked to cell morphology [40, 43]. Morphological parameters of living U251 cells expressing different GFAP isoforms were determined by measuring the cell surface area and the perimeter. From these parameters, we calculated the form factor, as described in the methods section. Forty cells were analyzed per experiment in five independent experiments. GFAPδ expression caused the cells to become rounder (0.56 ± 0.006; mean ± SEM) in comparison with the control (0.49 ± 0.005, *p* = 0.009) (Fig. 6a). We also observed significant differences in perimeter (Fig. 6b) and area (Fig. 6c) between cells expressing GFAPδ and GFAPα.

To assess whether the morphological changes we observed were due to changes in cell-extracellular matrix interaction, the sizes of focal adhesions were assessed by phosphorylated paxillin stainings in cells plated on a laminin substrate. In both GFAPα and GFAPδ expressing cells, the size of the focal adhesion was increased (mCherry: 1.13 μm^2^ ± 0.02; GFAPα: 1.34 μm^2^ ± 0.03; GFAPδ: 1.37 μm^2^ ± 0.03 (Fig. 6e, f). There was, however, no transcriptional regulation of the main laminin-binding integrins expressed in these cells (data not shown).

### GFAP overexpression does not affect cell motility or cell proliferation

**Fig. 7:**
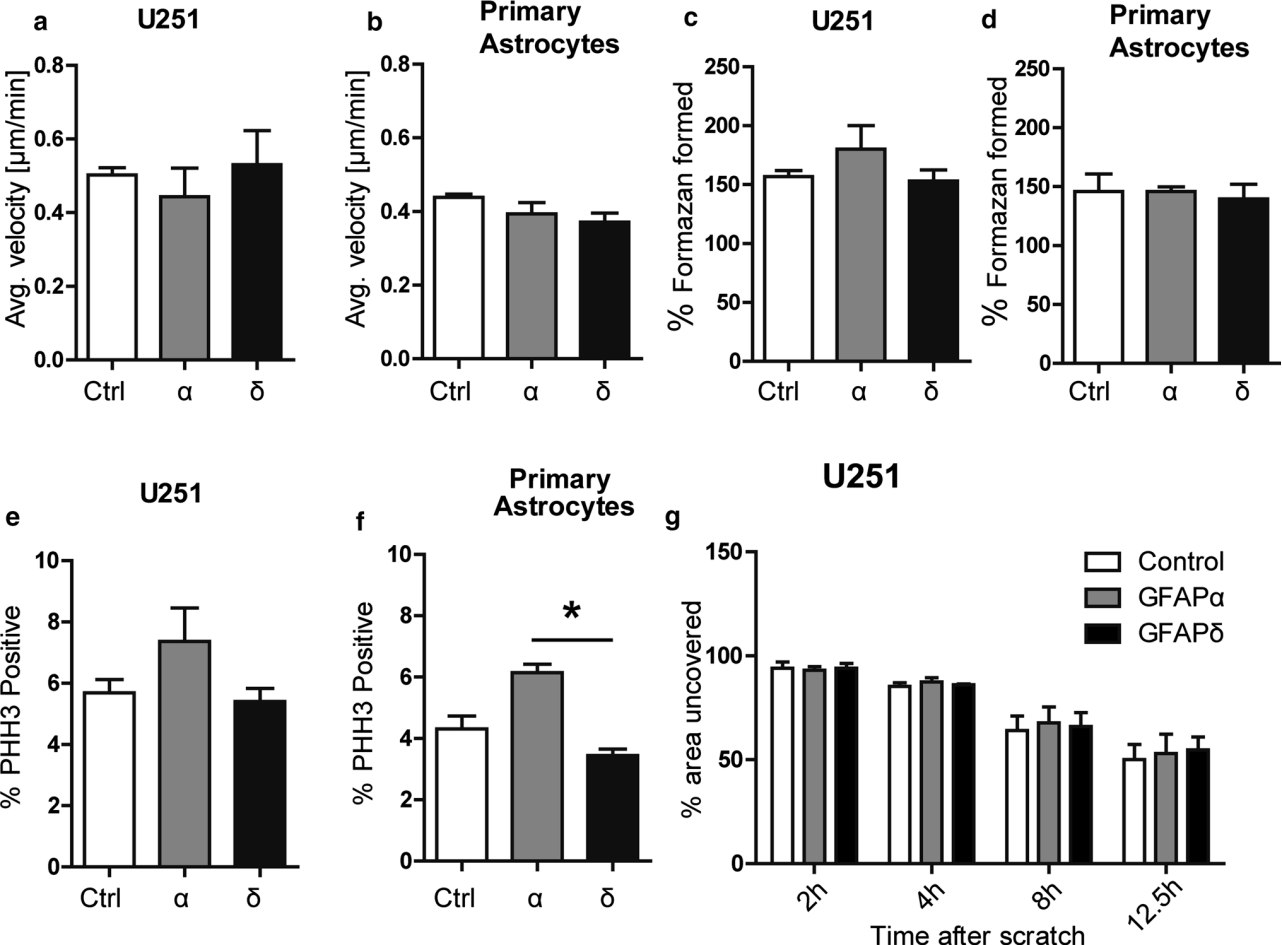
Migration and proliferation is not affected by GFAPδ. **a** Single cell motility was measured as the average velocity in μm/min of a single cell, in a sequence of images, which were taken overnight. Average velocity did not differ significantly between GFAPα, GFAPδ, and control cells (*p* = 0.9) (*n* = 3) in U251 cells or in **b** primary astrocytes (*p* = 0.15) (*n* = 3). **c** Proliferation was measured by metabolic conversion of MTT, which was measured by absorbance of light. The absorbance at *t* = 24 h was put at 100 %. There was no significant difference between GFAPδ, GFAPα, and control (*p* = 0.56) (*n* = 3) at 48 h in U251 cells or **d** primary astrocytes (*p* = 0.56) (*n* = 3). **e** Cells were stained for PHH3, indicating dividing cells. The average percentage of dividing cells per condition at one time point is not significantly different in cells with GFAPα, GFAPδ, and control in U251 cells (*p* = 0.21) (*n* = 4) (**f**). In primary astrocytes, there was a significant difference in proliferation between GFAPα and GFAPδ (*p* = 0.04) (*n* = 3). **g** Scratch assays were done by scratching a monolayer of cells and measuring the area uncovered by cells, over time. The area of the scratch at *t* = 0 was put at 100 %. The *bars* represent the area where there are no cells at different time points. There was no significant difference (*p* = 0.7) (*n* = 3) between GFAPα, GFAPδ, and the control after 12.5 h. All graphs show mean with SEM

Focal adhesions are tightly regulated during cell migration, and GFAP has been linked to in vitro migration by others [4, 43]. To check for changes in cell motility, we analyzed single cell motility and scratch wound healing speed. Single cell motility assays were performed on U251 cells expressing GFAPα, GFAPδ, or mCherry. Cells were seeded on PLL coated glass coverslips, and thirty cells were analyzed per experiment in 3 independent experiments. There were no statistically significant differences in average velocity between U251 cells expressing GFAPδ (0.53 µm/min ± 0.09), GFAPα (0.44 µm/min ± 0.08), or control (0.50 µm/min ± 0.02) (*p* = 0.9) (Fig. 7a). Similar results were found in a wound healing assay where a monolayer of cells was scratched and the wound healing speed was measured over time (Fig. 7g). Wound healing speed of U251 cells expressing GFAPα (52.9 % ± 9.3), GFAPδ (54.7 % ± 6.2), or the control (50 % ± 7.3) was not different at 12.5 h (*p* = 0.7). To study the effects of GFAP isoform expression on primary cell motility instead of on tumor cells, single cell motility assays were also done on primary human astrocytes. Again, we found no statistically significant difference between cell motility of cells expressing GFAPα (0.39 ± 0.03), GFAPδ (0.37 ± 0.02), and control cells (0.43 ± 0.009) (*p* = 0.15) (Fig. 7b).

Since GFAPδ is highly expressed in proliferating astrocytes, i.e., neurogenic astrocytes [7, 8] and astrocytoma cells [12, 14], we determined the effect of GFAPδ expression on proliferation by performing a 3‐(4,5‐Dimethylthiazol‐2‐yl)‐2,5‐diphenyltetrazolium bromide (MTT) assay [47]. We observed no significant difference between proliferation of GFAPδ (153 % ± 9.2), GFAPα (180 % ± 19.8), and control (mCherry) (156 % ± 5.3) (*p* = 0.56) over a period of 48 h in U251 cells (Fig. 7c). The MTT assay in primary astrocytes also did not show significant differences between GFAPα (146 % ± 3.8), GFAPδ (139 % ± 12.5), and control (146 % ± 14.7) (*p* = 0.56) (Fig. 7d). To confirm these results, proliferation was also assessed by staining for the proliferation marker Phospho histone H3 (PHH3). U251 cells transduced with the GFAP isoforms were plated, fixed 48 h later, and stained for PHH3 (Fig. 7e). We observed no significant difference (*p* = 0.21) in the percentage of PHH3 positive cells between GFAPα (7.3 % ± 1.1), GFAPδ (5.4 % ± 0.4), and control (5.6 % ± 0.4). PHH3 staining in primary astrocytes did, however, show a significant difference between GFAPα (6.1 % ± 0.3) and GFAPδ (3.4 % ± 0.2) (*p* = 0.04) (Fig. 7f). Taken together, these data show that GFAPδ expression did not alter cell proliferation or motility, while GFAPα led to a slightly higher proliferation rate compared with GFAPδ.

## Discussion

Astrocytes and astrocytoma cells tightly regulate the expression of at least 10 different GFAP isoforms [6]. GFAPα and GFAPδ are the two most highly expressed isoforms. The functional consequences of changes in the IF network in astrocytes and astrocytoma cells are still elusive. In this study, we have investigated the effect of GFAPδ on cell proliferation, migration, motility, and shape but also the intracellular effects on IF network dynamics. We here show that GFAPα and GFAPδ have different intrinsic dynamic properties, but that expression of GFAPδ does not affect cell proliferation or migration when there is a collapse of the IF network.

## Dynamic properties

GFAPδ is assembly compromised by itself [16] and causes a collapse of the whole IF network. We here show that even before a collapse, GFAPδ changes the dynamics of the the IF network. Dynamic properties of IF proteins differ between IF family members [50–53]. These differences are likely due to phosphorylation or structural properties that affect the assembly. The latter is for instance reflected in higher FRAP recovery half time values by the nuclear lamins (~140 min) [53, 54], which forms IgG-like folds during assembly [55]. In contrast, GFAP and vimentin, which are both located in the cytoplasm, have half times between 1 and 5 min [51, 52]. In this study, we showed that GFAPδ incorporates and dissociates slower from an IF network than GFAPα. Since the C-terminal tail is the only difference between GFAPα and GFAPδ, the differences in dynamics must be due to this specific sequence. IF protein motility and exchange are mediated through interactions with microtubules and actin to regulate transport [50], and IF protein phosphorylation to regulate filament stability [25, 56, 57]. In the part of the C-terminus, where GFAPα and GFAPδ differ, the same amount of putative phosphorylation sites are present (7 residues). However, the position of these residues is different, possibly leading to a different availability of phosphorylatable residues in the protein tertiary structure. In the tail of GFAPδ is a coil 2B binding site, which lacks in the GFAPα tail [16]. It has been proposed by Nielsen and Jorgensen [16] that this domain results in a gain of coiled-coil binding activity of GFAPδ, which might explain the slower dissociation of GFAPδ from an IF network. Earlier experiments have shown that ablation of the whole C-terminal tail of IF type III proteins inhibits IF assembly [38, 58], but phosphorylation of vimentin at the C-terminal side did not affect its assembly [59]. Mutant GFAP isoforms, with mutated phosphorylation sites, will help to elucidate the effects of GFAP phosphorylation on network dynamics.

## Effect on proliferation, motility, and migration

In this study, we showed that a GFAPδ-induced collapse of the IF network results in a reorganization of the whole IF network. This is in contrast with a knockout or knockdown of GFAP, which does not severely affect vimentin or nestin localization [40]. GFAP, as well as other IFs, has been linked to changes in proliferation and astrocyte motility, but it has to be noted that the studies on the role of GFAP in cell proliferation are inconclusive. A GFAP knockdown has been shown to lead to an increase in proliferation in some studies [4, 60], but not in others [61]. On the other hand, astrocyte cultures of transgenic mice overexpressing human GFAPα showed a decrease in proliferation [62]. Since GFAPδ is expressed in cycling cells in the human brain [8, 12, 14], we expected an effect of GFAPδ on cell proliferation. Unexpectedly, we observed that not the expression of GFAPδ, but an enhanced expression of GFAPα resulted in a significant increase in proliferation of primary astrocytes. We detected a similar trend in the U251 astrocytoma cells. Our data show that the increase in proliferation is caused by the mere increase in GFAPα and that there is no direct role of GFAPδ in cell proliferation in cells with a collapsed IF network. Lowering the ratio of GFAPα:GFAPδ making sure that there is still an intact network also did not change cell proliferation in vitro [40].

Several studies have found that a knockdown or knockout of GFAP increases cell motility [4, 43, 63]. In addition, we recently showed that a specific knockdown of GFAPα leads to a reduced motility in astrocytoma cells [40].Here, we show that an overexpression of GFAPα or GFAPδ has no effect on cell motility, even if the IF network is collapsed by high GFAPδ levels. Regulation of motility by GFAP is rather complex. Mutations in the rod domain of GFAP that causes collapses of the network, and thus mimics our GFAPδ condition, have been shown to increase cell motility. In contrast, mutations in the tail domain of GFAP had no effect on motility [64, 65]. GFAPα and GFAPδ only differ in the C-terminal tail, thus this might explain why we do not see differences in cell motility.

## Effect on ECM interaction

GFAP has been linked to cell morphological changes in astrocytes in vitro, where a correlation was observed between the level of GFAP expression and the number of cell protrusions [4, 41–43, 61]. In reactive gliosis, the production of GFAP as well as other IF proteins is upregulated. This results in a more pronounced, GFAP-positive, IF network [66–68]. Here, we show that the collapse of the IF network due to GFAPδ expression resulted in more round cells with longer focal adhesions in vitro. This change resembles the morphological change in astrocytes devoid of IFs in vitro [4, 41, 43, 61]. Thus, an intact IF network is important for the formation or stabilization of processes of cells. In our earlier study, we showed, however, that a pan-GFAP knockdown in U373 cells did not result in a rounder morphology [40]. This is probably due to the presence of an intact vimentin and nestin IF network, which is lacking in the cells with a GFAPδ-induced collapse.

The change we observed in cell morphology in cells with a GFAPδ-induced collapsed IF network shows that the IF network distribution per se can affect the shape of the cell. The way GFAPδ alters shape is likely to occur through integrins. Integrins are the main linkers between the extracellular matrix and the cytoskeleton in vivo and, together with other proteins, they form the focal adhesions. Focal adhesions can be present in different sizes and maturation states, ranging from small structures, of less than 1 µm, to larger focal adhesions. Focal adhesion size is dependent on actomyosin generated tension [69, 70]. Indeed, interactions between the IF protein vimentin and mature focal adhesions have been shown to be essential for proper cell spreading [71]. Vimentin can regulate adhesion and focal contact size under shear stress [72] and controls cell adhesion strength through plectin and β3 integrins [73]. Thus, GFAP could have a similar function in adhesion of astrocytes to the ECM and in this way alter cell shape. Our data confirm a direct effect of the IF constellation on focal adhesions and cell–matrix interactions, as both GFAP isoforms increase focal adhesion size. Since only GFAPδ expressing cells showed altered morphology, this implies a difference in the effect GFAPα and GFAPδ has on the functionality of focal adhesions. This might partly be due to differences in production of ECM molecules, such as laminin, as we have shown before [40]. Although the exact interaction of GFAP with focal adhesions is still elusive, interactions between other IFs and focal adhesions have been described, as is nicely reviewed by Leube et al. [74].

## Conclusion

To summarize, we have shown that a GFAPδ-induced collapse of the IF network has a profound effect on IF network morphology, increases focal adhesion size, and changes the IF network dynamics, without altering astrocyte motility or proliferation. Although GFAPδ is expressed in more proliferative cell types with a higher migration potential, GFAPδ itself does not directly influence proliferation or migration when there is a collapse of the IF network. The changes in IF network dynamics could hold clues to GFAP isoform specific functions.

## Electronic supplementary material

Below is the link to the electronic supplementary material.

**Sup Fig.** **1.** GFAP isoform specific overexpression models. GFAP mRNA levels were determined in U251 cells (a, b) and in primary human astrocytes (d, e), 7 days after transduction with GFAPα or GFAPδ lentiviral constructs. The transduced GFAP isoforms gave the highest expression in both U251 (n = 4) and primary human astrocytes (n = 3). The overall expression levels of GFAP are higher in primary human astrocytes compared with U251 cells. Overexpression is confirmed at protein level with Western blot. In U251 cells, the specific upregulation was shown with isoform specific antibodies, the GFAP c-term antibody distinctively recognizes GFAPα and the hGFAPδ antibody recognizes GFAPδ. The band in the control condition was recognized by a pan GFAP antibody and reflects the endogenous presences of mostly GFAPα. Due to less sensitivity of the C-term antibody, this band is not visible when blots are stained with this antibody. (f) Endogenous expression levels of GFAP isoforms in U251 astrocytoma cell lines and primary human astrocytes show that GFAPα and GFAPδ are the most abundant isoforms expressed. (TIFF 1722 kb)

**Sup Fig.** **2.** Cytoskeleton in primary human astrocytes with a collapsed IF network. Primary human astrocytes transduced with GFAPα, control plasmid, and GFAPδ, as indicated by the fluorescent reporter, showed that microtubules (a) and actin filaments (b) were not co-collapsing with the IF network. Microtubules and actin filaments were still present throughout the whole cells in GFAPδ transduced cells. Hst = Hoechst. Scale bar represents 20 μm. * indicate the transduced cells in the GFAPα condition of 2b. (JPEG 1918 kb)

**Sup Fig.** **3.** mRNA expression of IFs in GFAP isoform expressing U251 cells. In U251 cells transduced with GFAP isoforms, mRNA was measured for the other IFs. There is no significant regulation of endogenous GFAPα (a), GFAPδ (b), or vimentin (d mRNA and e protein). The nestin mRNA expression (c) was significantly regulated only in cells ectopically expressing GFAPα protein (p = 0.03). (TIFF 976 kb)

**Sup Fig.** **4.** Incorporation of GFP–GFAP isoforms in a collapsed IF network. U251MG cells expressing GFAPδ showed a collapse of the IF network, as seen in a and b by analyzing GFAP and vimentin fluorescence. When co-expressed with GFAPδ, both GFP–GFAPα and GFP–GFAPδ were incorporated into the collapse (arrows). GFP fluorescence co-localized with GFAP and vimentin immunostaining, showing that the dynamics measured in these experiments reflect GFAP in a collapsed network. Cells transfected with GFP–GFAPδ showed a similar collapsed structure as the GFP–GFAPα cells. Scale bar represents 20 µm. (TIFF 5999 kb)

**Sup Fig.** **5.** GFP–GFAP incorporates into the endogenous IF network. (a–c) U251 cells transfected with GFP–GFAPα or GFP–GFAPδ showed incorporation of the fusion protein into the endogenous IF network. Cells were fixed 24 h after transfection and stained for GFP, GFAP, and vimentin. (a) After 24 h, GFP–GFAPα transfected cells showed the presence of GFP in the endogenous spread out network, indicating that this fusion protein assembled with endogenous IF proteins. After 24 h, GFP–GFAPδ transfected cells showed both cells with spread out networks (b), as well as with collapsed IF networks (c). In both cases, the GFP fusion protein co-localized with the endogenous IF network. Scale bar represents 20 μm. (JPEG 2056 kb)

**Sup Movie 1.** U343MG cells were transfected with GFP–GFAPδ and imaged for 48 h. As the expression of GFP–GFAPδ comes up the GFAP network becomes faintly visible. As the expression increases, the GFAP network collapses and condensates near the nucleus. The collapsed network remains motile within the cell. Scale bar represents 20 μm. (AVI 10,327 kb)

**Sup Movie 2.** This movie is a zoom in on one of the cells shown in Sup Movie 1 where U343MG cells were transfected with GFP–GFAPδ and imaged for 48 h. Here, it is clear to see that small parts of fluorescent GFP–GFAP move around in the cytoplasm before they condensate near the nucleus. Scale bar represents 20 μm. (AVI 1349 kb)
