# Supplementary figures and images for: GFAP isoforms control intermediate filament network dynamics, cell morphology, and focal adhesions
Source: Cell Mol Life Sci. 2016 May 3;73(21):4101–20. doi: 10.1007/s00018-016-2239-5 (PMC5043008; doi:10.1007/s00018-016-2239-5)

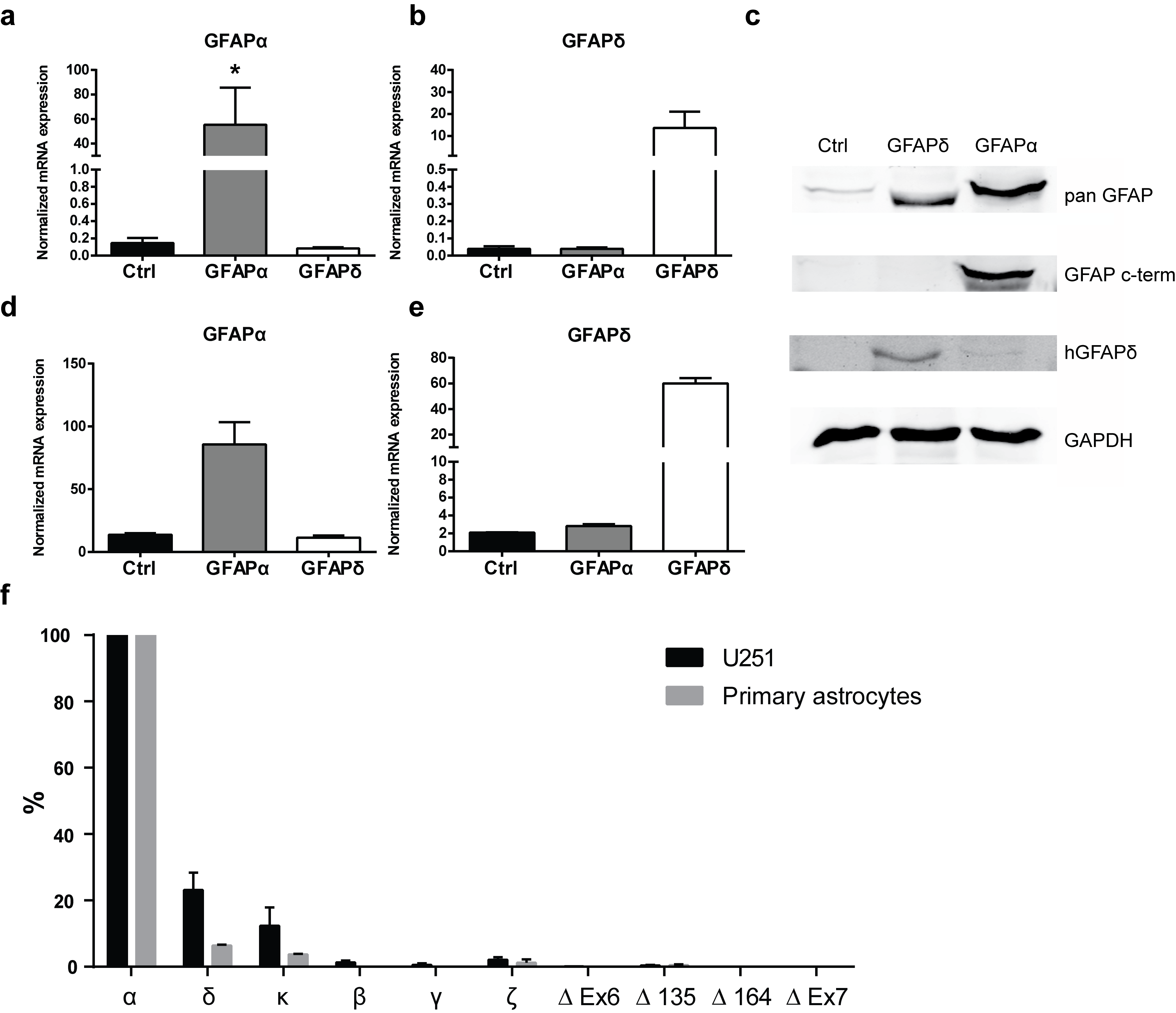

Supplement: Supplementary file 1 — Sup Fig. 1. GFAP isoform specific overexpression models. GFAP mRNA levels were determined in U251 cells (a, b) and in primary human astrocytes (d, e), 7 days after transduction with GFAPα or GFAPδ lentiviral constructs. The transduced GFAP isoforms gave the highest expression in both U251 (n = 4) and primary human astrocytes (n = 3). The overall expression levels of GFAP are higher in primary human astrocytes compared with U251 cells. Overexpression is confirmed at protein level with Western blot. In U251 cells, the specific upregulation was shown with isoform specific antibodies, the GFAP c-term antibody distinctively recognizes GFAPα and the hGFAPδ antibody recognizes GFAPδ. The band in the control condition was recognized by a pan GFAP antibody and reflects the endogenous presences of mostly GFAPα. Due to less sensitivity of the C-term antibody, this band is not visible when blots are stained with this antibody. (f) Endogenous expression levels of GFAP isoforms in U251 astrocytoma cell lines and primary human astrocytes show that GFAPα and GFAPδ are the most abundant isoforms expressed. (TIFF 1722 kb) [file 18_2016_2239_MOESM1_ESM.tif]

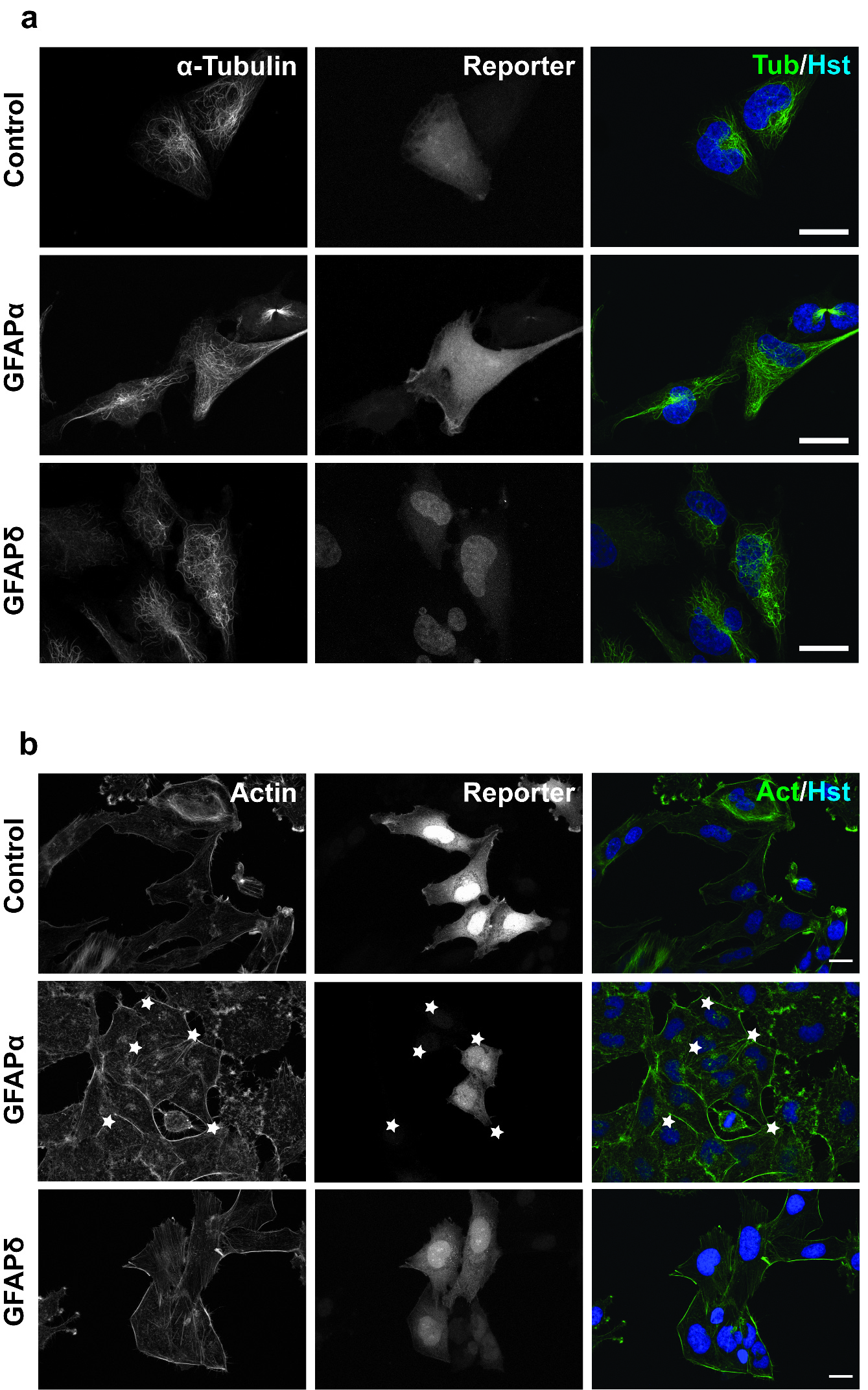

Supplement: Supplementary file 2 — Sup Fig. 2. Cytoskeleton in primary human astrocytes with a collapsed IF network. Primary human astrocytes transduced with GFAPα, control plasmid, and GFAPδ, as indicated by the fluorescent reporter, showed that microtubules (a) and actin filaments (b) were not co-collapsing with the IF network. Microtubules and actin filaments were still present throughout the whole cells in GFAPδ transduced cells. Hst = Hoechst. Scale bar represents 20 μm. * indicate the transduced cells in the GFAPα condition of 2b. (JPEG 1918 kb) [file 18_2016_2239_MOESM2_ESM.jpg]

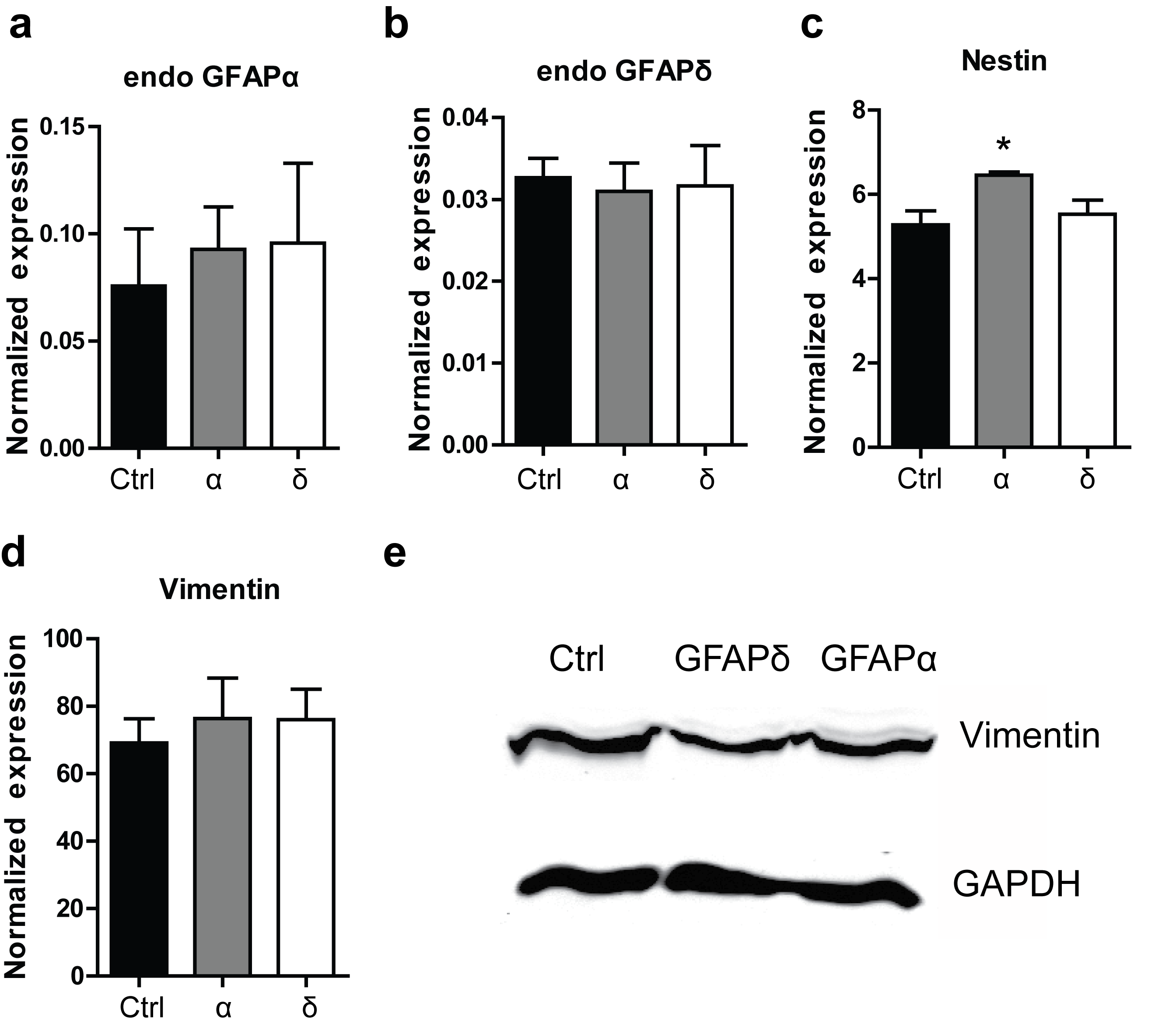

Supplement: Supplementary file 3 — Sup Fig. 3. mRNA expression of IFs in GFAP isoform expressing U251 cells. In U251 cells transduced with GFAP isoforms, mRNA was measured for the other IFs. There is no significant regulation of endogenous GFAPα (a), GFAPδ (b), or vimentin (d mRNA and e protein). The nestin mRNA expression (c) was significantly regulated only in cells ectopically expressing GFAPα protein (p = 0.03). (TIFF 976 kb) [file 18_2016_2239_MOESM3_ESM.tif]

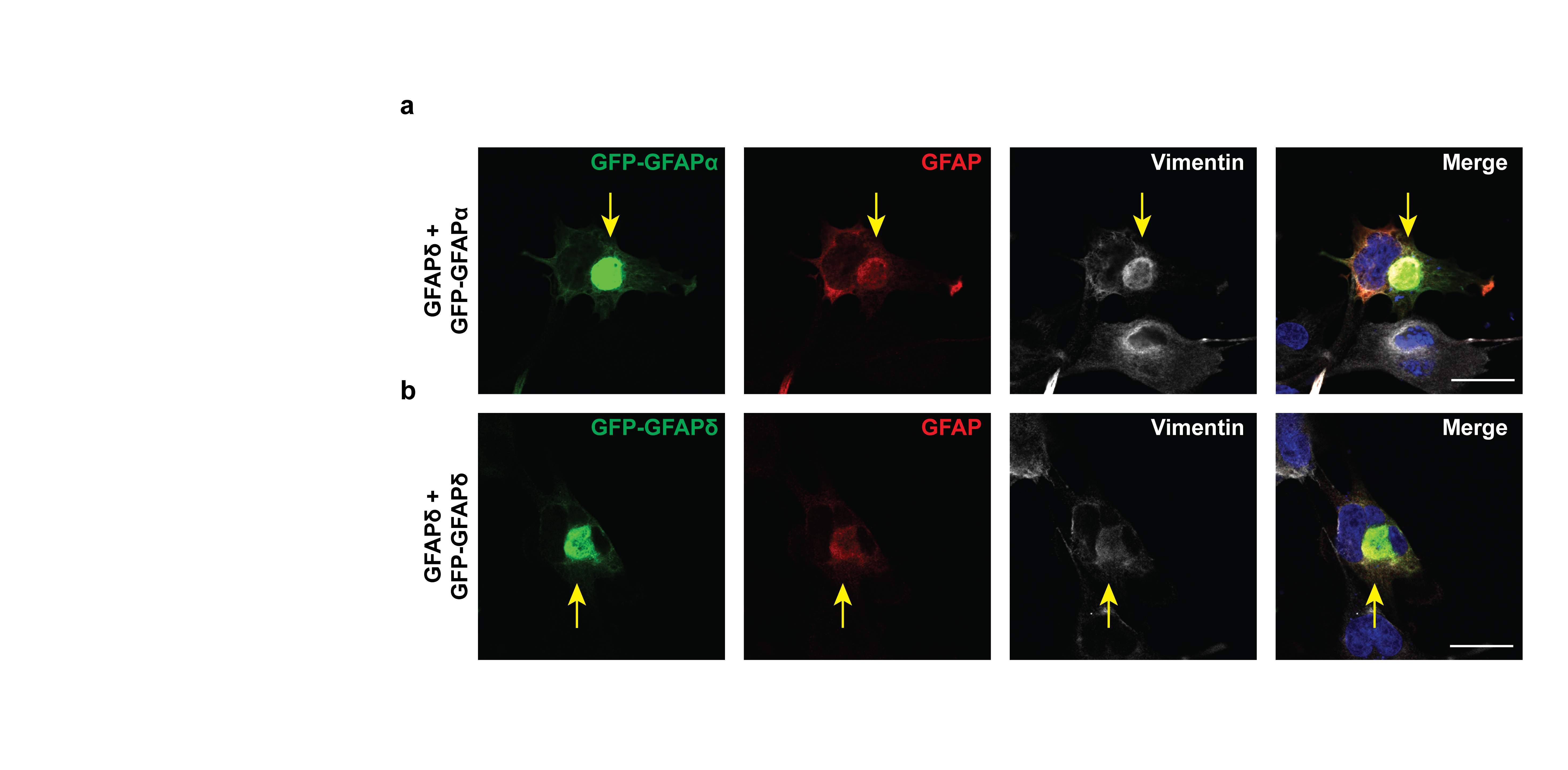

Supplement: Supplementary file 4 — Sup Fig. 4. Incorporation of GFP–GFAP isoforms in a collapsed IF network. U251MG cells expressing GFAPδ showed a collapse of the IF network, as seen in a and b by analyzing GFAP and vimentin fluorescence. When co-expressed with GFAPδ, both GFP–GFAPα and GFP–GFAPδ were incorporated into the collapse (arrows). GFP fluorescence co-localized with GFAP and vimentin immunostaining, showing that the dynamics measured in these experiments reflect GFAP in a collapsed network. Cells transfected with GFP–GFAPδ showed a similar collapsed structure as the GFP–GFAPα cells. Scale bar represents 20 µm. (TIFF 5999 kb) [file 18_2016_2239_MOESM4_ESM.tif]

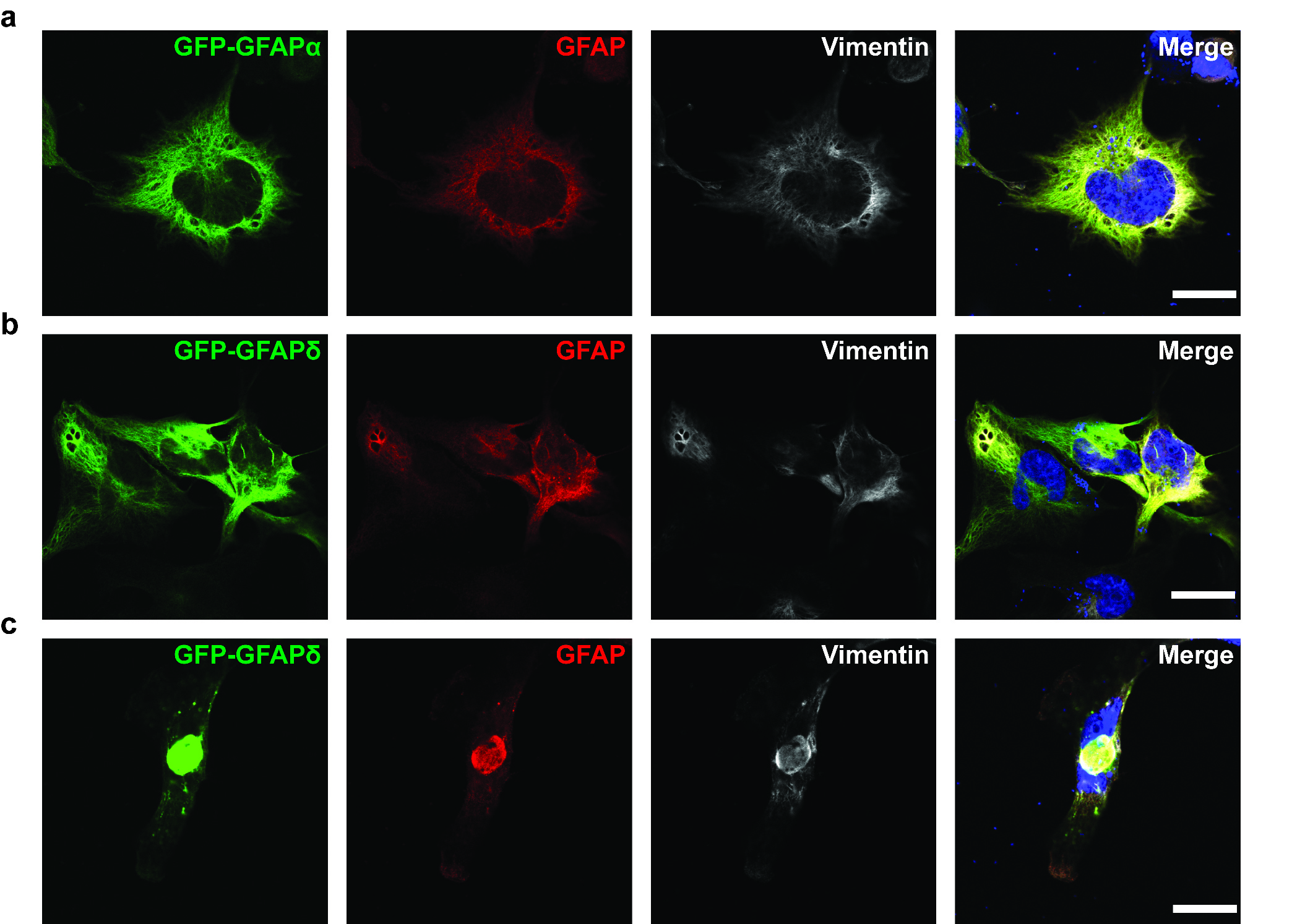

Supplement: Supplementary file 5 — Sup Fig. 5. GFP–GFAP incorporates into the endogenous IF network. (a–c) U251 cells transfected with GFP–GFAPα or GFP–GFAPδ showed incorporation of the fusion protein into the endogenous IF network. Cells were fixed 24 h after transfection and stained for GFP, GFAP, and vimentin. (a) After 24 h, GFP–GFAPα transfected cells showed the presence of GFP in the endogenous spread out network, indicating that this fusion protein assembled with endogenous IF proteins. After 24 h, GFP–GFAPδ transfected cells showed both cells with spread out networks (b), as well as with collapsed IF networks (c). In both cases, the GFP fusion protein co-localized with the endogenous IF network. Scale bar represents 20 μm. (JPEG 2056 kb) [file 18_2016_2239_MOESM5_ESM.jpg]
